# Hormone-dependent control of developmental timing through regulation of chromatin accessibility

**DOI:** 10.1101/gad.298182.117

**Published:** 2017-05-01

**Authors:** Christopher M. Uyehara, Sabrina L. Nystrom, Matthew J. Niederhuber, Mary Leatham-Jensen, Yiqin Ma, Laura A. Buttitta, Daniel J. McKay

**Affiliations:** 1 Department of Biology, The University of North Carolina at Chapel Hill, Chapel Hill, North Carolina, 27599, USA;; 2 Department of Genetics, The University of North Carolina at Chapel Hill, Chapel Hill, North Carolina, 27599, USA;; 3 Curriculum in Genetics and Molecular Biology, The University of North Carolina at Chapel Hill, Chapel Hill, North Carolina, 27599, USA;; 4 Integrative Program for Biological and Genome Sciences, The University of North Carolina at Chapel Hill, Chapel Hill, North Carolina, 27599, USA;; 5 Department of Molecular, Cellular, and Developmental Biology, University of Michigan, Ann Arbor, Michigan 48109, USA

**Keywords:** temporal gene regulation, open chromatin, ecdysone, pioneer transcription factor, genomics

## Abstract

Specification of tissue identity during development requires precise coordination of gene expression in both space and time. Spatially, master regulatory transcription factors are required to control tissue-specific gene expression programs. However, the mechanisms controlling how tissue-specific gene expression changes over time are less well understood. Here, we show that hormone-induced transcription factors control temporal gene expression by regulating the accessibility of DNA regulatory elements. Using the *Drosophila* wing, we demonstrate that temporal changes in gene expression are accompanied by genome-wide changes in chromatin accessibility at temporal-specific enhancers. We also uncover a temporal cascade of transcription factors following a pulse of the steroid hormone ecdysone such that different times in wing development can be defined by distinct combinations of hormone-induced transcription factors. Finally, we show that the ecdysone-induced transcription factor E93 controls temporal identity by directly regulating chromatin accessibility across the genome. Notably, we found that E93 controls enhancer activity through three different modalities, including promoting accessibility of late-acting enhancers and decreasing accessibility of early-acting enhancers. Together, this work supports a model in which an extrinsic signal triggers an intrinsic transcription factor cascade that drives development forward in time through regulation of chromatin accessibility.

## Abstract (precis)

Uyehara et al. show that hormone-induced transcription factors control temporal gene expression by regulating accessibility of DNA regulatory elements. Using the *Drosophila* wing, they demonstrate that temporal changes in gene expression are accompanied by genome-wide changes in chromatin accessibility at temporal-specific enhancers.

A defining feature of metazoan development is the organization of cells into tissues. The physiological function of a given tissue is determined by the identity of its constituent cells as well as their arrangement within the tissue. As a result, building tissues during development requires precise spatial control of gene expression over extended periods of time. Whereas many of the genes required for the development of different cell and tissue types have been identified, the mechanisms through which spatial information is coordinated with temporal information remain incompletely understood.

Spatially, a select and conserved group of transcription factors, sometimes termed “master” transcription factors, often specifies the distinct identities of different cell and tissue types (Mann and Carroll 2002; Mullen et al. 2011). Genetic studies from a range of organisms show that loss of function of a given master transcription factor can result in the loss of a given cell type or tissue. Conversely, ectopic expression of a given master transcription factor can result in transformation of identities. Hence, master transcription factors are major determinants of cell fate. Consistent with their importance in development, the dysregulation of master transcription factors is associated with a range of diseases. Thus, understanding the mechanisms through which these factors function is an important goal in biomedical research.

One proposed mechanism to explain the distinctive power of master transcription factors is that they control where other transcription factors bind in the genome by regulating chromatin accessibility (Fakhouri et al. 2010; Mullen et al. 2011; Pham et al. 2013). In vivo, DNA is wrapped around histone proteins to make nucleosomes, the basic unit of chromatin. Due to their tight association with DNA, nucleosomes act as barriers to transcription factor binding. For a given transcription factor to bind DNA, a nucleosome must be moved or evicted, creating a site of “open” or “accessible” chromatin. Several lines of evidence support an important role for chromatin accessibility in transcription factor targeting in the genome. Chief among these are the observations that only a small fraction of transcription factor DNA-binding motifs is occupied at a given point in time (Li et al. 2008) and that many sites of transcription factor binding do not contain a recognizable DNA-binding motif (Kvon et al. 2012). Thus, regulation of chromatin accessibility plays a potentially pivotal role in controlling cell identity by determining where transcription factors can bind in the genome and hence the sets of genes that are expressed.

If nucleosomes prevent transcription factors from accessing DNA, then how do transcription factors come to occupy their binding sites? Biochemical studies have identified a class of transcription factors termed “pioneer” factors that have the unique ability to bind nucleosomal DNA and subsequently enable binding by other transcription factors (Zaret and Mango 2016). The prototype pioneer factor is FoxA1, a master regulator of liver development (Lee et al. 2005). FoxA1 has also been shown to play an important role in controlling targeting of the estrogen and androgen receptors in breast and prostate cancer cells, respectively (Jozwik and Carroll 2012). More recently, the master transcription factors of embryonic stem cell identity—Oct4, Sox2, and Klf4—were shown to have pioneering activity during induction of pluripotency in induced pluripotency stem (iPS) cells (Soufi et al. 2015). While pioneers have the potential to be pivotal regulators of gene expression programs, much remains to be learned about their function. For example, it is not clear why they exhibit pioneering activity in only a subset of the cells in which they are expressed. Pioneers also may not be the only factors that control chromatin accessibility. Other transcription factors can work together to compete nucleosomes off DNA, consistent with earlier in vitro work on transcription factor binding to nucleosomal templates (Taylor et al. 1991).

In addition to spatial control, gene expression patterns are also temporally regulated in development. For example, in a variety of animals, neural stem cells produce daughter cells with distinct identities at different times of development to create the vast diversity of neurons and glia found in the nervous system (Kohwi and Doe 2013). In *Drosophila* embryos, an intrinsic cascade of transcription factor expression specifies the distinct temporal identities of neural stem cell progeny (Isshiki et al. 2001). A similar mechanism using a different transcription factor cascade diversifies neural identities in the *Drosophila* larval brain (Erclik et al. 2017). In contrast to stem cell line-ages, coordinating the timing of gene expression across fields of cells, such as a tissue, often involves the use of secreted signals. For example, thyroid hormone controls the initiation and progression of metamorphosis in frogs (Shi 2013), whereas the sex hormones control the development of secondary sex traits during adolescence in mammals (Romeo 2003).

In *Drosophila* and other insects, developmental timing is controlled by the steroid hormone ecdysone (Ashburner 1990; Thummel 2002). Secreted by the prothoracic gland at stereotypical stages of development, ecdysone travels through the hemolymph to reach target tissues, where it binds to its receptor, the ecdysone receptor (EcR) (King-Jones and Thummel 2005). Like other nuclear hormone receptors, EcR is a transcription factor that differentially regulates gene expression in the presence and absence of ligand. Studies initially performed in the larval salivary gland revealed that, upon binding ecdysone, EcR activates transcription of a set of early genes, many of which are transcription factors (Ashburner 1990). The early gene products then work with EcR to activate a set of late genes, which encode the proteins that mediate the physiological response to hormone signaling (e.g., the glue proteins made by the salivary gland that adhere the pupa to a substrate during metamorphosis). Transcriptional profiling from a diverse collection of cell lines showed that the response to ecdysone is both widespread and highly cell type-specific (Stoiber et al. 2016). Mapping of hormone-responsive enhancers in cultured cells recently revealed that tissue-specific responses to ecdysone are influenced by motif content in DNA regulatory elements (Shlyueva et al. 2014). Despite these efforts, the precise mechanisms through which ecdysone signaling controls temporal-specific gene expression in *Drosophila* remain elusive.

To ask how spatial and temporal information are integrated by regulatory DNA during specification of tissue identities, we recently performed open chromatin profiling at two stages of *Drosophila* appendage development (McKay and Lieb 2013). In flies, the distinct identity of each appendage is determined by the expression of different master transcription factors with different DNA-binding domains. For example, leg identity is determined by the homeodomain transcription factor *Distalless* and the zinc finger transcription factor *Sp1* (Estella and Mann 2010). In contrast, dorsal appendage identities, including the wing and haltere, are specified by *vestigial* and its TEA domain-containing DNA-binding partner, *scalloped* (Halder et al. 1998). Despite the differences in master transcription factor identities between these tissues and contrary to our expectations, we found that the open chromatin profiles in wings, legs, and halteres are nearly the same, with the exception of the master regulator loci themselves, which exhibit differential accessibility between the appendages (McKay and Lieb 2013). The similarity in appendage open chromatin profiles indicates that the master transcription factors are not the sole determinants of chromatin accessibility, if they do so at all. This leaves the question of which factors are responsible for controlling chromatin accessibility in the appendages.

One clue to the potential identity of these factors came from comparisons of open chromatin profiles between appendages at different stages of development. We found that the different adult appendages shared very similar open chromatin profiles, similar to our findings from an earlier stage of appendage development, the third instar imaginal discs. However, open chromatin profiles of the adult appendages were markedly different from those of the imaginal discs. This indicates that a coordinate change in chromatin accessibility occurs during appendage development and also suggests that passage through developmental time has a greater impact than cell lineage on chromatin accessibility. Because the appendages are not in physical contact with each other inside developing flies, we reasoned that a systemic signal, such as ecdysone, contributes to control of chromatin accessibility.

Here, we examined the mechanisms controlling temporal gene regulation in *Drosophila*. Using a time course of wing development that encompasses the transition between larval and pupal stages, we used RNA sequencing (RNA-seq) to show that gene expression is temporally dynamic as wings differentiate and undergo the complex morphogenetic events that create the adult appendage. We then carried out open chromatin profiling and transgenic reporter analysis to show that these changes in gene expression are accompanied by genome-wide changes in the accessibility of temporal-specific transcriptional enhancers. Finally, we used ChIP-seq (chromatin immunoprecipitation [ChIP] combined with high-throughput sequencing) and loss-of-function analyses to show that the ecdysone-induced transcription factor E93 is required to drive the normal sequence of chromatin accessibility changes. Importantly, E93 is required for not only increasing the accessibility of late-acting enhancers but also decreasing the accessibility of early-acting enhancers. Together, these findings demonstrate that E93 specifies temporal identity by directly regulating accessibility of temporal-specific transcriptional enhancers. More broadly, this work helps to explain how hormone signaling can influence tissue-specific gene expression programs to drive development forward in time.

## Results

### Gene expression is temporally dynamic in pupal wings

**Figure 1.**
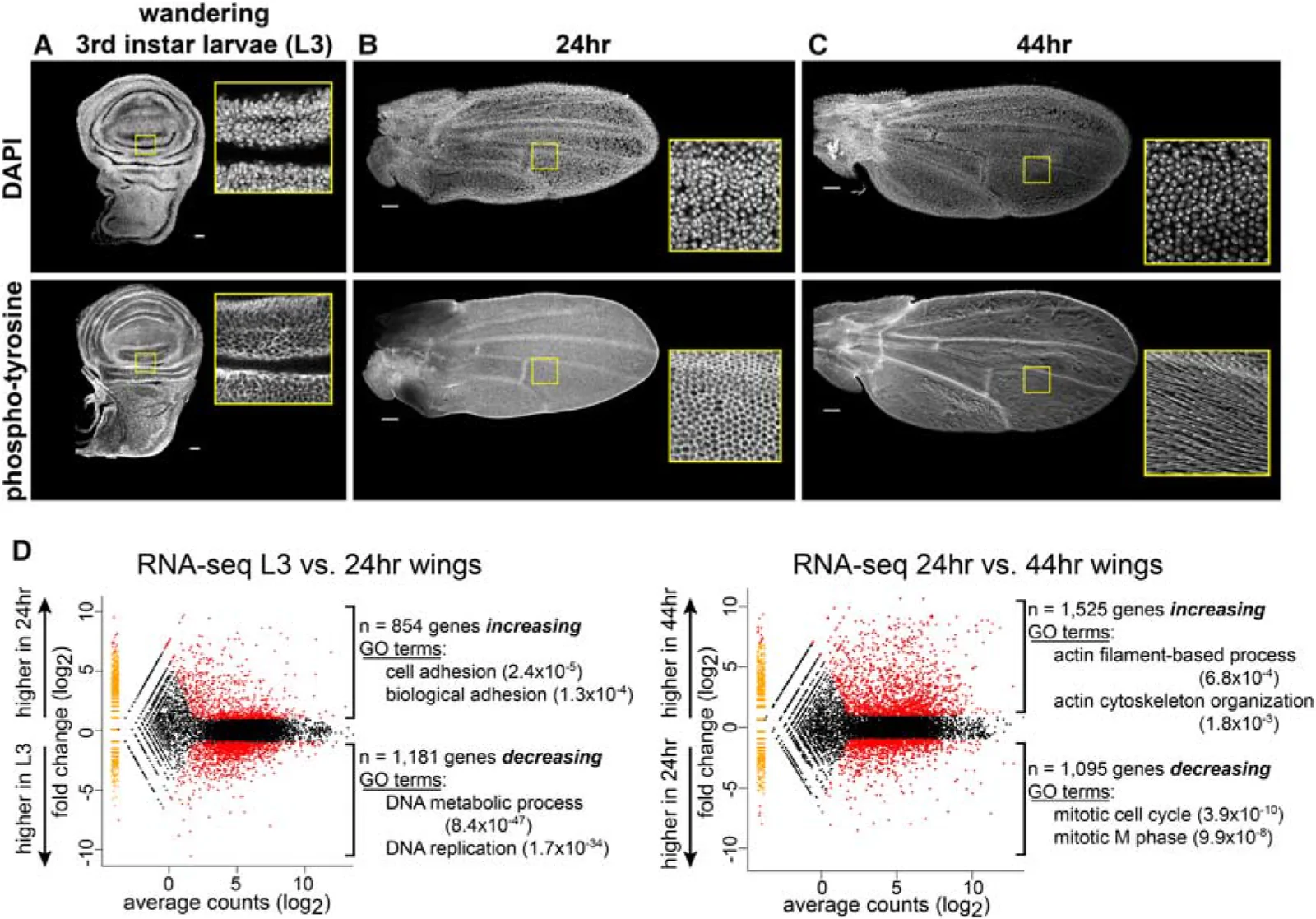
Gene expression is temporally dynamic in pupal wings. (*A*–*C* ) Immunostaining of wings from three developmental time points. DAPI (*top* row) and phospho-tyrosine (*bottom* row) label nuclei and cell membranes, respectively. (*D*) MA plots of RNA-seq signal in annotated genes for consecutive time points. Differentially expressed genes are colored red. The top two GO terms for differentially expressed genes are indicated with *P*-values in parentheses. Bars, 50 µm.

To examine the mechanisms underlying temporal regulation of gene expression, we focused on the early stages of *Drosophila* pupal wing development. By the end of larval development (Fig. 1A), the wing disc consists of ∼50,000 cells, cell fates along the proximal–distal axis have been patterned, and precursors of adult structures such as wing veins and sensory organs are being specified (Cohen 1993). During the next 2 d of pupal development (Fig. 1B, C), cell fates continue to be more finely determined, while the wing undergoes a final round of cell division (Guo et al. 2016). This time is also characterized by dramatic morphological changes at both the tissue and cellular levels: Changes in cell shape drive eversion of the wing pouch, and changes in cell adhesion allow the apposed dorsal and ventral surfaces of the wing epithelium to form the upper and lower layers of the wing blade. Cytoskeletal changes also result in extrusion of the cell membrane to produce a single cuticular hair (trichome) from each wing blade epithelial cell (Fig. 1C, bottom row; Adler et al. 2000). Not surprisingly, these developmental changes are associated with widespread changes in gene expression. To quantify these changes, we performed RNA-seq on wing discs dissected from wandering third instar larvae (Fig. 1A, “L3”) and wings dissected from flies 24 h (Fig. 1B, “24 h”) and 44 h (Fig. 1C, “44 h”) after puparium formation (Guo et al. 2016). Pairwise comparisons between successive time points revealed thousands of genes both increasing and decreasing between each time point (edgeR false discovery rate [FDR] <0.05; fold change greater than or equal to twofold for expressed genes) (Fig. 1D; Supplemental Table S1). Gene ontology (GO) analysis showed enrichment for biological processes known to occur at these times (Huang da et al. 2009; Supek et al. 2011). For example, genes increasing between L3 and 24 h include those involved in cell adhesion (*P*-value 2.4 × 10^−5^), whereas genes increasing between 24 and 44 h include those involved in actin regulation (*P* value-6.8 × 10^−4^). Conversely, genes decreasing between L3 and 24 h include those involved in DNA replication (*P*-value 1.7 × 10^−34^), whereas genes decreasing between 24 and 44 h include those involved in mitosis (*P*-value 3.9 × 10^−10^) (Fig. 1D; Supplemental Fig. S1). Thus, the first 2 d of pupal wing development are marked by temporally dynamic changes in gene expression.

### Open chromatin profiles are temporally dynamic in pupal wings

**Figure 2.**
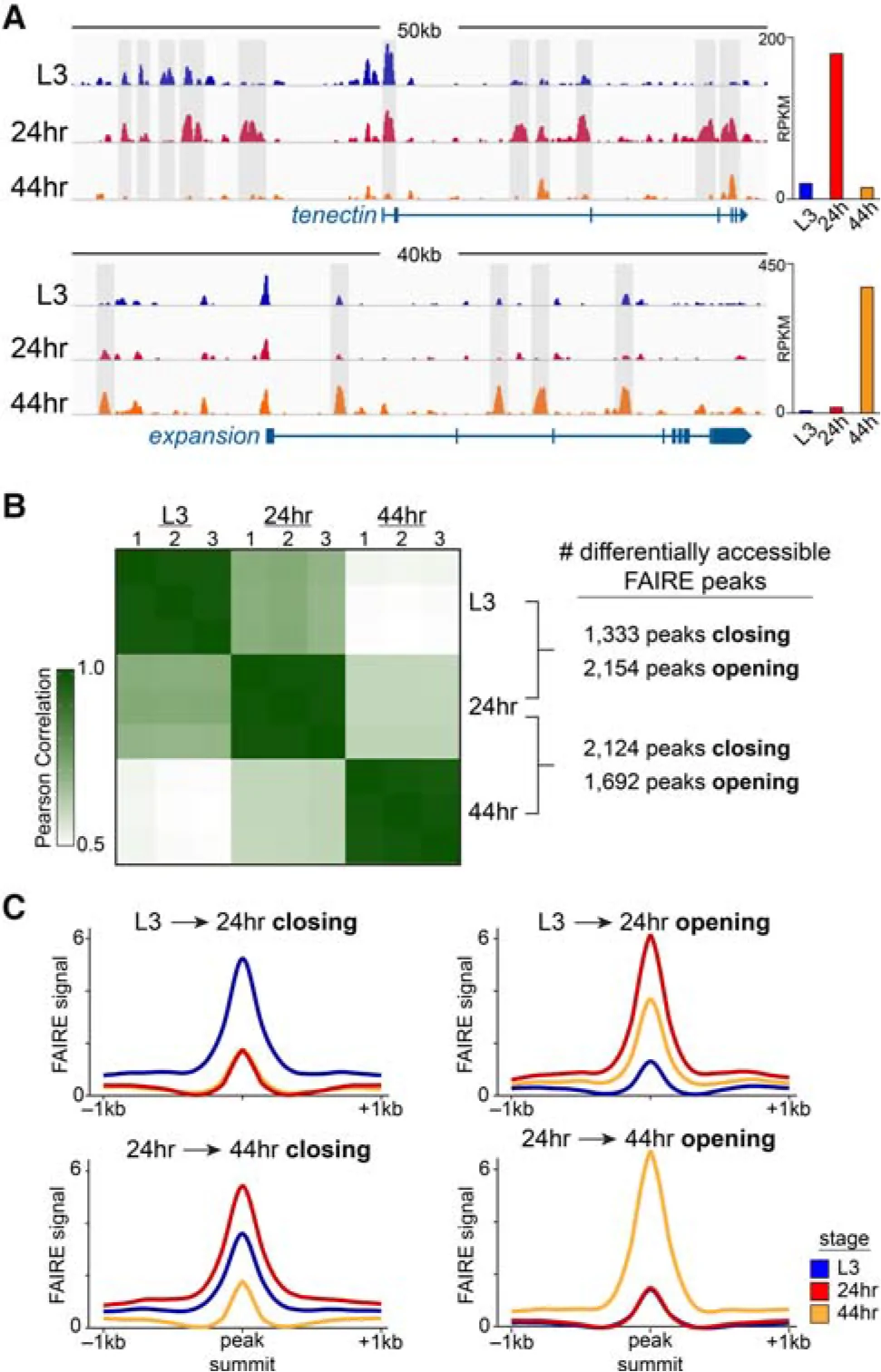
Open chromatin profiles are temporally dynamic in pupal wings. (*A*) Browser shots of FAIRE-seq signal (*Z*-score) at the *tnc* and *expansion* loci. Temporally dynamic open chromatin sites are highlighted with gray shading. Bar plots show the RNA-seq signal for each gene over time. (*B*) Heat map of the Pearson correlation coefficients between FAIRE-seq replicates. The number of differentially accessible FAIRE peaks out of the top 7699 peaks for each consecutive time point is shown. (*C* ) Line plots of the average FAIRE-seq signal across all categories of differentially accessible FAIRE peaks. The L3 signal is shown in blue, 24 h is shown in red, and 44 h is shown in orange.

We next sought to examine the mechanisms underlying the temporal changes in gene expression that we observed in pupal wings. Due to the competition between transcription factors and nucleosomes for DNA binding, methods that identify nucleosome-depleted regions, also known as open chromatin sites, can be used as a proxy to identify sites of transcription factor binding in the genome. To map open chromatin sites genome-wide, we performed FAIRE-seq (formaldehyde-assisted identification of regulatory elements [FAIRE] combined with sequencing) (Giresi et al. 2007) on L3, 24-h, and 44-h wings (Fig. 2). We found that open chromatin profiles in early pupal wings are highly dynamic between time points, with changes in open chromatin occurring at genes that change expression between time points (Supplemental Fig. S2A). For example, the *tenectin* gene (*tnc*), which encodes a constituent of the extra-cellular matrix that binds α-PS2 integrin (Fraichard et al. 2010), exhibits multiple open chromatin changes between L3, 24-h, and 44-h wings (Fig. 2A). These changes coincide with a strong increase in *tnc* expression between L3 and 24-h wings (Fig. 2A). Similarly, the *expansion* locus, which encodes a protein involved in chitin biosynthesis (Sobala and Adler 2016), contains multiple open chromatin sites that become accessible specifically between 24 h and 44 h. The timing of this chromatin opening coincides with an increase in *expansion* RNA levels (Fig. 2A) and the production of chitin by wing epidermal cells during cuticle secretion at this stage of development (Sobala and Adler 2016). At the genome-wide level, we found that approximately one-third of open chromatin sites are temporally dynamic (Fig. 2B; Supplemental Fig. S2B). Of the top 7699 FAIRE peaks from each time point (corresponding to a MACS2 *Q*-value of 40), 2154 sites increase and 1333 sites decrease in accessibility between L3 and 24-h wings (edgeR FDR <0.05; fold change greater than or equal to two-fold) (Fig. 2B). Similarly, 1692 peaks increase and 2124 peaks decrease in accessibility between 24-h and 44-h wings (Fig. 2B; Supplemental Fig. S2C). Henceforth, we refer to sites that decrease in accessibility between successive time points as “closing” and sites that increase in accessibility between successive time points as “opening.” We found that the great majority of these temporally dynamic open chromatin sites (78%–89%) is located distal to gene promoters (Supplemental Fig. S2D). Finally, plots of the average FAIRE signal in temporally dynamic open chromatin indicate that many temporally dynamic open chromatin sites are used transiently in development. For example, sites closing between L3 and 24 h tend to stay closed, and sites opening between 24 h and 44 h tend to be closed at L3 (i.e., prior to 24 h) (Fig. 2C). Thus, the dynamic gene expression exhibited by early pupal wings co-incides with dynamic changes in chromatin accessibility.

### Temporally dynamic open chromatin sites correspond to temporal-specific transcriptional enhancers

Open chromatin sites are highly correlated with functional DNA regulatory element activity (McKay and Lieb 2013). Our findings above suggest that temporally dynamic open chromatin sites may be transiently used promoter-distal enhancers in pupal wings. To test this directly, we cloned open chromatin sites from three genes for use in transgenic reporter assays. These sites were chosen because they exhibit temporally dynamic accessibility, and the neighboring genes are required for proper wing development. Candidate enhancers were cloned into reporter constructs and integrated into the genome as single copies via ΦC31-mediated site-specific recombination. Altogether, we cloned six temporally dynamic open chromatin sites. Each of these six sites corresponds to a temporally regulated transcriptional enhancer. We discuss each of them in turn.

**Figure 3.**
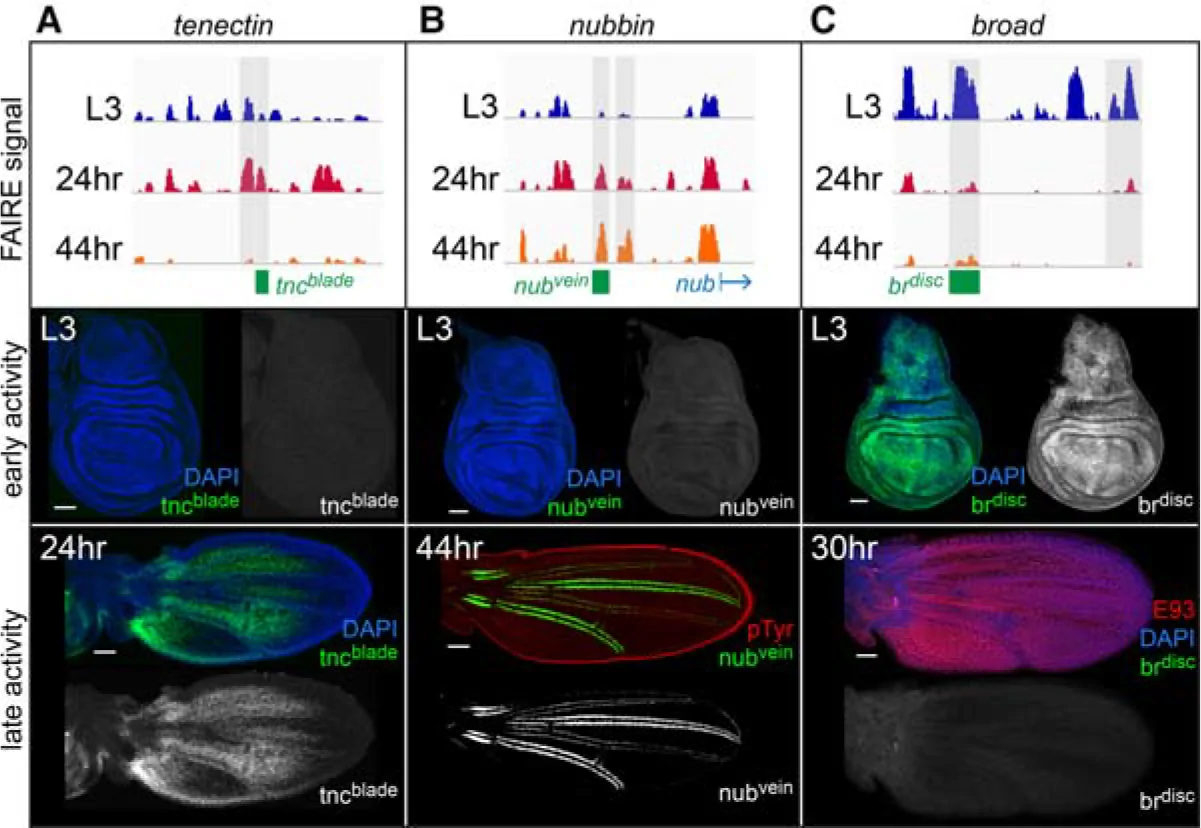
Temporally dynamic open chromatin corresponds to temporal-specific enhancer activity. (*Top* row) Browser shots of FAIRE-seq signal from the *tnc* (*A*), *nub* (*B*), and *broad* (*C* ) loci, with cloned regions indicated by gray boxes, and depicted enhancers indicated by green boxes. (*Middle* and *bottom* rows) Immunostaining of reporter activity in wings at the indicated early and late time points. Enhancer activity is in green. Bars, 50 µm. Additional time points are shown in Supplemental Figure S3.

We first examined two candidate enhancers from the *tnc* locus. As mentioned above, *tnc* encodes an extracellular matrix protein involved in cell adhesion. Consistent with a role for *tnc* in mediating adhesion between the dorsal and ventral surfaces of the wing pouch, RNAi-mediated knockdown of tnc results in defects in wing morphology (Fraichard et al. 2010). We cloned two temporally dynamic open chromatin sites located ∼40 kb up-stream of the *tnc* promoter. Our FAIRE-seq data show that these sites increase in accessibility between the L3 and 24-h time points and subsequently decrease in acces-sibility between 24 h and 44 h (Fig. 3A; Supplemental Figs. S3A, S4A). While neither reporter is active in L3 wing discs, there is activity in 24-h wings. We termed these the *tnc*^*blade*^ (blade) and *tnc*^*wv*^ (wing vein) enhancers. *tnc*^*blade*^ is active most strongly in the interveins between the first and second and between the fourth and fifth longitudinal veins and in cells near the proximal posterior margin. It is also active at lower levels in the intervein between the third and fourth longitudinal veins (Fig. 3A). *tnc*^*wv*^ is active most strongly near the first, fifth, and sixth longitudinal veins and at lower levels in the third longitudinal vein (Supplemental Fig. S4A). Thus, *tnc*^*blade*^ and *tnc*^*wv*^ are active in complementary domains of the 24-h pupal wing. Since *tnc* is expressed nearly ubiquitously at this stage of wing development, these open chromatin sites likely correspond to bona fide transcriptional enhancers that interpret different spatial inputs.

We next examined two candidate enhancers from the *nubbin* (*nub*) locus, which encodes a transcription factor required for proximal-distal axis and vein development in wings (Cifuentes and Garcia-Bellido 1997). The cloned candidate enhancers are located ∼5 and 6.5 kb upstream of the *nub* promoter. Our FAIRE-seq data show that these sites progressively increase in accessibility between the L3 and 44-h time points (Fig. 3B; Supplemental Figs. S3B, S4B). Immunofluorescence experiments show that a reporter carrying the distal site is not active in L3 wing discs. By 44 h, it shows strong activity in the L3 and L5 wing veins and weaker activity in the L2 and L4 veins (Fig. 3B). We thus designated this as the *nub*^*vein*^ enhancer. Consistent with the *nub*^*vein*^ activity pattern, hypomorphic *nub* alleles show defects in wing vein development (Cifuentes and Garcia-Bellido 1997). Immunostaining of the more proximal site, which we named *nub*^*margin*^, shows reporter activity near the wing margin and the posterior cross-vein of 44-h wings (Supplemental Fig. S4B), again consistent with defects observed in *nub* hypomorphic alleles (Cifuentes and Garcia-Bellido 1997). Thus, temporally dynamic open chromatin sites identify functionally relevant enhancers with temporal-specific activity.

Last, we examined two candidate enhancers from the *broad* (*br*) locus, which encodes a family of transcription factors active in third instar and early prepupal tissues, including the wing (Kiss et al. 1988; Guo et al. 2016). Using our FAIRE-seq data, we identified open chromatin sites at the *br* locus that are accessible in L3 wing discs but sub-sequently decrease in accessibility by 24 h and 44 h (Fig. 3C; Supplemental Figs. S3C, S4C). These candidate enhancers were cloned upstream of *GAL4* to allow for flexibility in the reporters used. Crossing these *GAL4* drivers to flies containing *UAS-GFP* revealed that both open chromatin sites are transcriptional enhancers active in L3 wing discs. The *br*^*disc*^ enhancer is located ∼40 kb up-stream of the *br* promoter. Similar to Br protein, *br*^*disc*^ is active nearly ubiquitously in wing imaginal disc epithelial cells, with higher levels along the anterior–posterior and dorsal–ventral boundaries in the wing pouch (Fig. 3C). We next sought to determine whether the decrease in accessibility of *br*^*disc*^ between L3 and 24 h coincides with a decrease in enhancer activity. Since there are a limited number of cell divisions in pupal wings, GFP signal can persist even after an enhancer turns off. Therefore, we used a destabilized GFP reporter, reasoning that in-creased GFP degradation may make the reporter more sensitive to the enhancer's activity state even if GAL4 persists. Consistent with the timing of *br*^*disc*^ closing, we found that it shuts off between L3 and 24 h (Fig. 3C). Thus, the timing of *br*^*disc*^ closing coincides with the timing of it turning off. We identified a second *br* enhancer, which we termed *br*^*ade*^ (Supplemental Fig. S4C). This enhancer is located ∼30 kb upstream of the *br* promoter and is active in L3 wing discs in a pattern similar to the adepithelial cells located in the notum of the wing. Like the *br*^*disc*^ enhancer, there is no sign of *br*^*ade*^ reporter activity in the wing blade by 24 h, consistent with expectations, since the adepithelial cells remain in the notum to form the indirect flight muscles. Notably, *br* is required for proper differentiation of these cells into adult muscles (Lovato et al. 2005). Together, these findings support the premise that temporally dynamic open chromatin sites correspond to temporal-specific transcriptional enhancers and that genes use different DNA regulatory elements to control their expression at different stages of development.

### A temporal cascade of ecdysone-induced transcription factors is expressed in pupal wings

**Figure 4.**
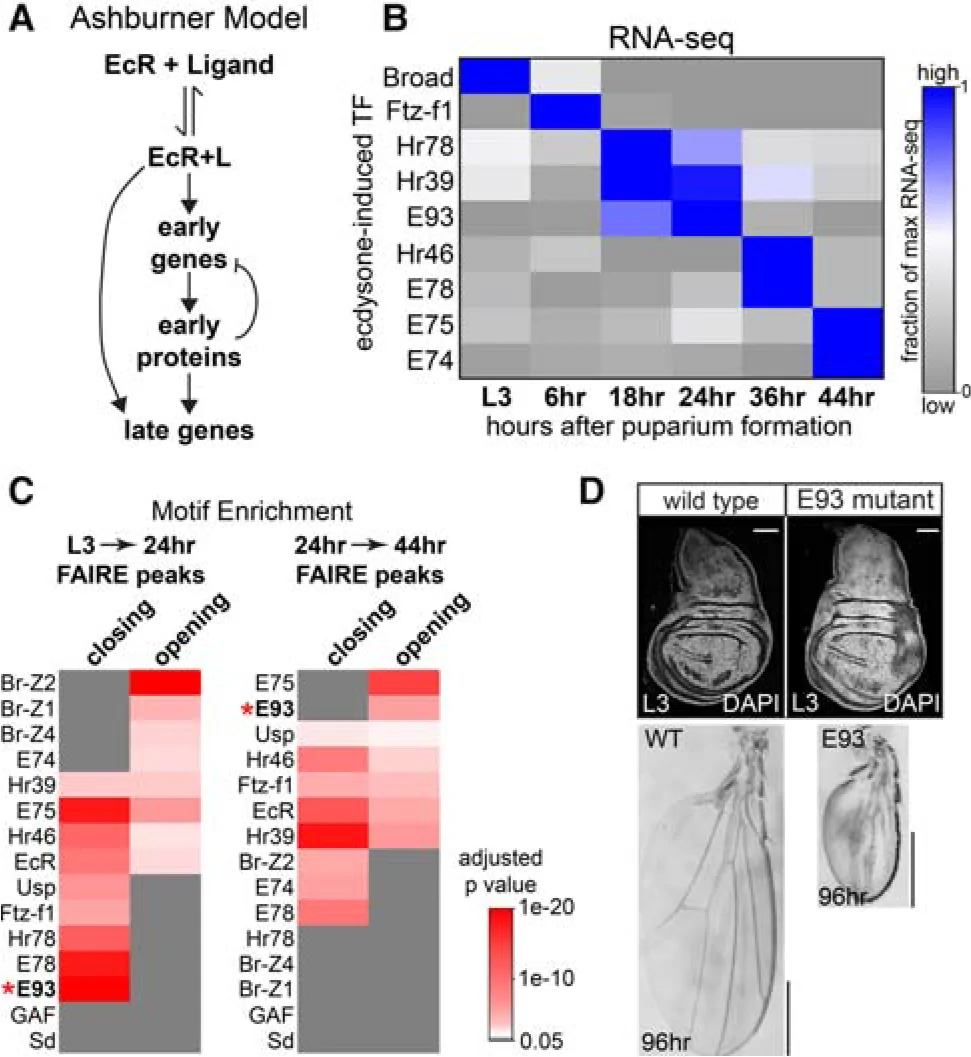
A temporal cascade of ecdysone-induced transcription factors in pupal wings. (*A*) Diagram of the Ashburner model of ecdysone signaling. (*B*) Heat map of gene expression values for selected ecdysone-induced genes across six stages of wing development, plotted as a fraction of the maximum expression value. Blue shows high expression, and gray shows low expression. (*C* ) Heat maps of DNA-binding site motif enrichment in dynamic FAIRE peaks for selected transcription factors. (*D*) DAPI stain of L3 wing discs (*top*) and bright-field images of 96- h wings (*bottom*) from wild-type (*left*) and *E93* mutants (*right*). Bars: *top*, 75 µm; *bottom*, 500 µm.

The above findings suggest that temporal changes in gene expression are driven by temporal changes in the accessibility of transcriptional enhancers. We next sought to identify factors that could be involved in controlling the accessibility of these enhancers. We reasoned that ecdysone signaling may be involved, since it controls developmental transitions in insects (Thummel 2001; King-Jones and Thummel 2005), and our previous work suggested that an extrinsic signal may coordinate temporal changes in chromatin accessibility between the appendages (McKay and Lieb 2013). We performed RNA-seq at six time points in pupal wings (Guo et al. 2016). Consistent with the Ashburner model of ecdysone signaling (Fig. 4A; Ashburner 1990), we observed a clear temporal cascade of ecdysone-induced transcription factor expression such that each time point in early pupal wing development can be defined by a distinct combination of these transcription factors (Fig. 4B; Supplemental Table S2). Moreover, the timing of each factor's expression coincides with the timing of its requirement in *Drosophila* development. For example, *br* is required for the transition from larval to prepupal stages (Kiss et al. 1988), and we found that it is expressed specifically at the L3 time point in wings. Likewise, *ftz-f1* is required for the transition from prepupal to pupal stages (Broadus et al. 1999), and we found that it is expressed specifically at the 6-h time point in wings. Finally, the transcription factor *E93* is expressed at the 18-h and 24-h time points, when it is required for bract development in pupal legs (Mou et al. 2012). Thus, a temporal cascade of ecdysone-induced transcription factors occurs in early pupal wings.

If ecdysone-induced transcription factors control chromatin accessibility, one may expect to find their DNA-binding motifs to be overrepresented in temporally dynamic open chromatin sites. To ask this question, we looked for enrichment of known DNA-binding motifs (Zhu et al. 2011) for a set of ecdysone-induced transcription factors (Supplemental Table S3) in temporally dynamic FAIRE peaks relative to temporally static FAIRE peaks. We observed significant enrichment (*P* <0.05) for multiple motifs in FAIRE peaks that open or close between successive time points (Fig. 4C). In contrast, we did not find any enrichment in temporally dynamic peaks for the motif of Scalloped (Sd), the DNA-binding partner of the wing master transcription factor Vestigial (Halder et al. 1998). We also looked for enrichment of the motif for GAGA factor (GAF), a transcription factor often associated with transcriptional enhancers and open chromatin sites (Fuda et al. 2015). We found that the GAF motif was enriched in both dynamic and static FAIRE peaks, thus causing no relative motif enrichment and suggesting that GAF is not responsible for the temporal dynamics. Together, these findings are consistent with a role for ecdysone-induced transcription factors in regulating temporally dynamic open chromatin sites.

The motif for the ecdysone-induced transcription factor E93 was strongly overrepresented in open chromatin sites that close between L3 and 24 h as well as those that open between 24 h and 44 h (Fig. 4C). We therefore sought to determine whether *E93* plays a role in wing development. E93 encodes a pipsqueak domain-containing transcription factor that was first identified as an ecdysone target required for autophagy of the larval salivary gland (Baehrecke and Thummel 1995; Lee et al. 2000). More recently, *E93* was shown to act as a competence factor for temporal-specific gene regulation in the pupal leg (Mou et al. 2012). Our RNA-seq data show that *E93* is transcribed at high levels in pupal wings at the 18- and 24-h time points (Fig. 4B). To ask whether *E93* is required for normal wing development, we compared the morphology of wild-type and *E93* mutant wings. At the L3 stage, wild-type and *E93* mutant wings are indistinguishable (Fig. 4D), consistent with expectations, since *E93* is not expressed at this time. At 24 h, when *E93* is expressed at high levels, *E93* mutant wings display defects in cell adhesion between the dorsal and ventral surfaces of the wing epithelium (data not shown). At 96 h, following the period of *E93* expression, *E93* mutant wings are dramatically smaller than wild-type wings, with significant defects in vein development (Fig. 4D). Thus, *E93* is essential for proper wing development, and the appearance of defects in *E93* mutants is commensurate with the timing of its expression in pupal wings.

### ChIP-seq reveals that E93 binds open chromatin sites in pupal wings

The above findings reveal that the temporal changes in gene expression that occur during pupal wing development coincide with temporal changes in the accessibility of thousands of open chromatin sites, many of which could be transcriptional enhancers. We next sought to examine the role of E93 in this process. As a first step, we performed ChIP-seq to identify sites in the genome to which E93 binds. We used an *E93* protein trap fly strain generated by the *Drosophila* Gene Disruption Project (Venken et al. 2011). In this strain, the endogenous *E93* gene has a transposon inserted within an intron that is shared by all annotated *E93* isoforms. The transposon carries an “artificial exon” cassette with the coding sequence for GFP and other epitope tags in the same reading frame as *E93*, flanked by splice acceptor and donor sites. Upon transcription and translation, an E93 fusion protein is expressed (referred to here as E93^GFSTF^) that can be immunoprecipitated with antibodies to GFP. Importantly, the *E93*^*GFSTF*^ chromosome complements a deletion en-compassing the *E93* locus [*Df(3R)93F*^*X2*^], demonstrating that the fusion protein is functional. Supporting this functionality, immunostaining of *E93*^*GFSTF*^ flies shows clear fusion protein expression in 24-h and 44-h wings (Supplemental Fig. S5).

**Figure 5.**
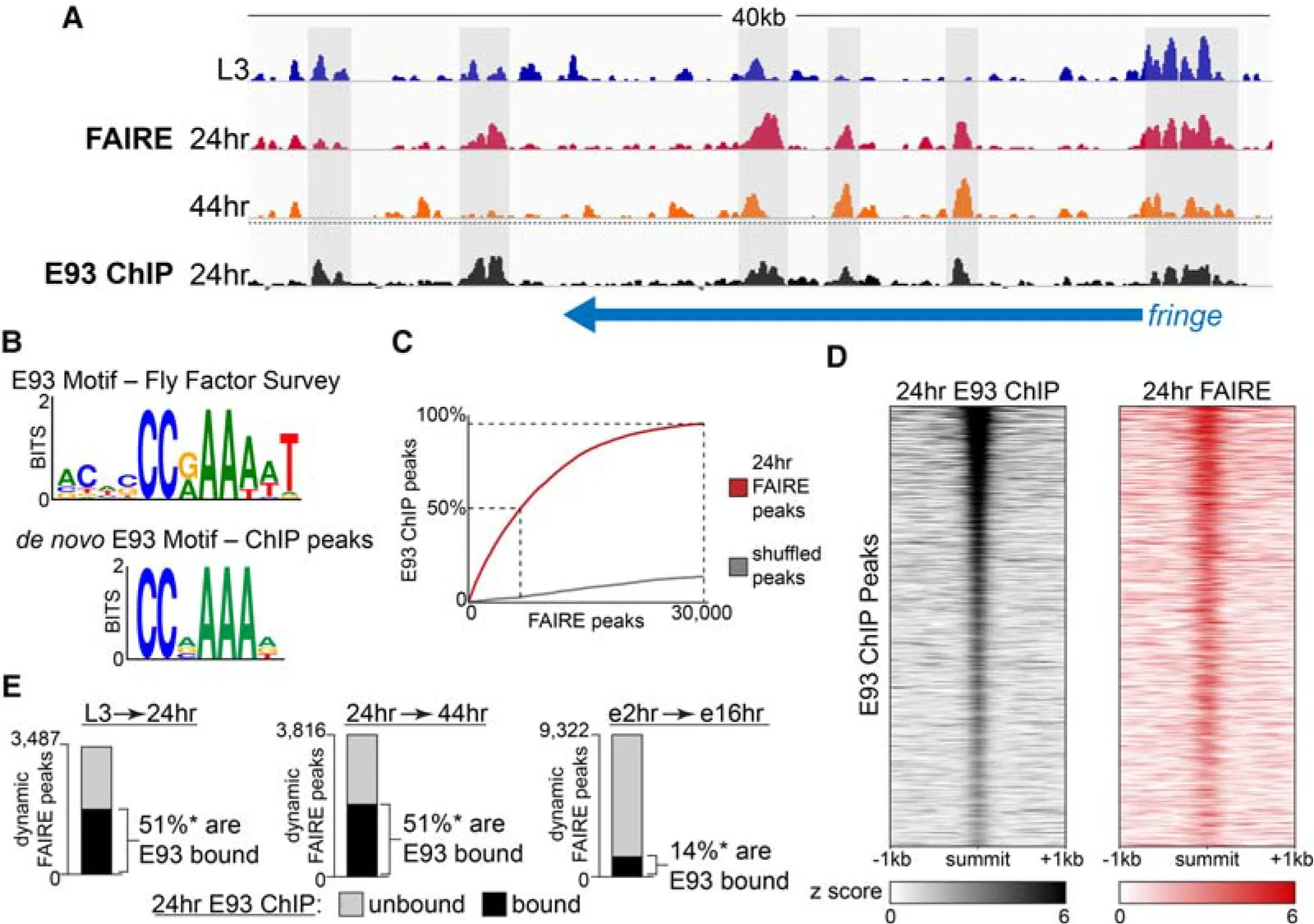
E93 binds temporally dynamic open chromatin. (*A*) Browser shot from the *fringe* locus showing FAIRE-seq and E93 ChIP-seq signals (*Z*-score) from pupal wings. (*B*) Position weight matrices comparing the E93 motif discovered in ChIP peaks with the known E93 motif. (*C*) Cumulative distribution plot of E93 ChIP peak overlap with 24-h FAIRE peaks (red line) relative to randomly shuffled FAIRE peaks (gray line). (*D*) Heat maps plotting E93 ChIP-seq and FAIRE-seq signals (*Z*-score) in E93 ChIP peaks from 24-h pupal wings. (*E*) Stacked bar plots showing the fraction of temporally dynamic FAIRE peaks (opening and closing) that overlap an E93 ChIP peak. (^∗^) Over-lap *P*-value <2.2 × 10^−16^ relative to temporally dynamic FAIRE peaks in embryos, Fisher's exact test.

We performed ChIP-seq for E93 on dissected 24-h pupal wings (Fig. 5A). Peak calling with MACS2 identified 8477 significantly bound sites genome-wide. De novo motif discovery analysis (Bailey 2011) identified a sequence enriched in E93 ChIP peaks that is very similar to an E93 motif derived from a bacterial one-hybrid screen for *Drosophila* transcription factors (Fig. 5B; Zhu et al. 2011), supporting the quality of the data. Overall, E93 binding corresponds well with 24-h open chromatin in pupal wings (Fig. 5C,D); 50% of E93 ChIP peaks are contained within the top 6225 24-h FAIRE peaks, and 96% of E93 ChIP peaks are contained within all 24-h FAIRE peaks (Fig. 5C). While there is good correspondence between E93 binding and open chromatin, not all 24-h FAIRE peaks are bound by E93 (Supplemental Fig. S6A), demonstrating that the E93 ChIP signal is specific and not simply an indirect consequence of open chromatin. This is further supported by differences in the distribution of E93 ChIP and 24-h FAIRE peak locations across the genome: E93 binds preferentially to promoter-distal sites in the genome, whereas FAIRE peaks overlap proximal promoter regions with greater frequency (Supplemental Fig. S6B). We next examined the relationship between E93 binding and temporally dynamic open chromatin in pupal wings. Fifty-one percent of FAIRE peaks that change in accessibility between L3 and 24 h are directly bound by E93 at 24 h. Likewise, 51% of FAIRE peaks that change in accessibility between 24 h and 44 h are directly bound by E93 at 24 h (Fig. 5E). In contrast, only 14% of temporally dynamic FAIRE peaks in early embryos (McKay and Lieb 2013) are bound by E93 in 24-h pupal wings. Thus, E93 directly binds a significant majority of open chromatin sites that change accessibility (opening or closing) between L3 and 44 h.

### E93 binding is required for temporally dynamic open chromatin changes

**Figure 6.**
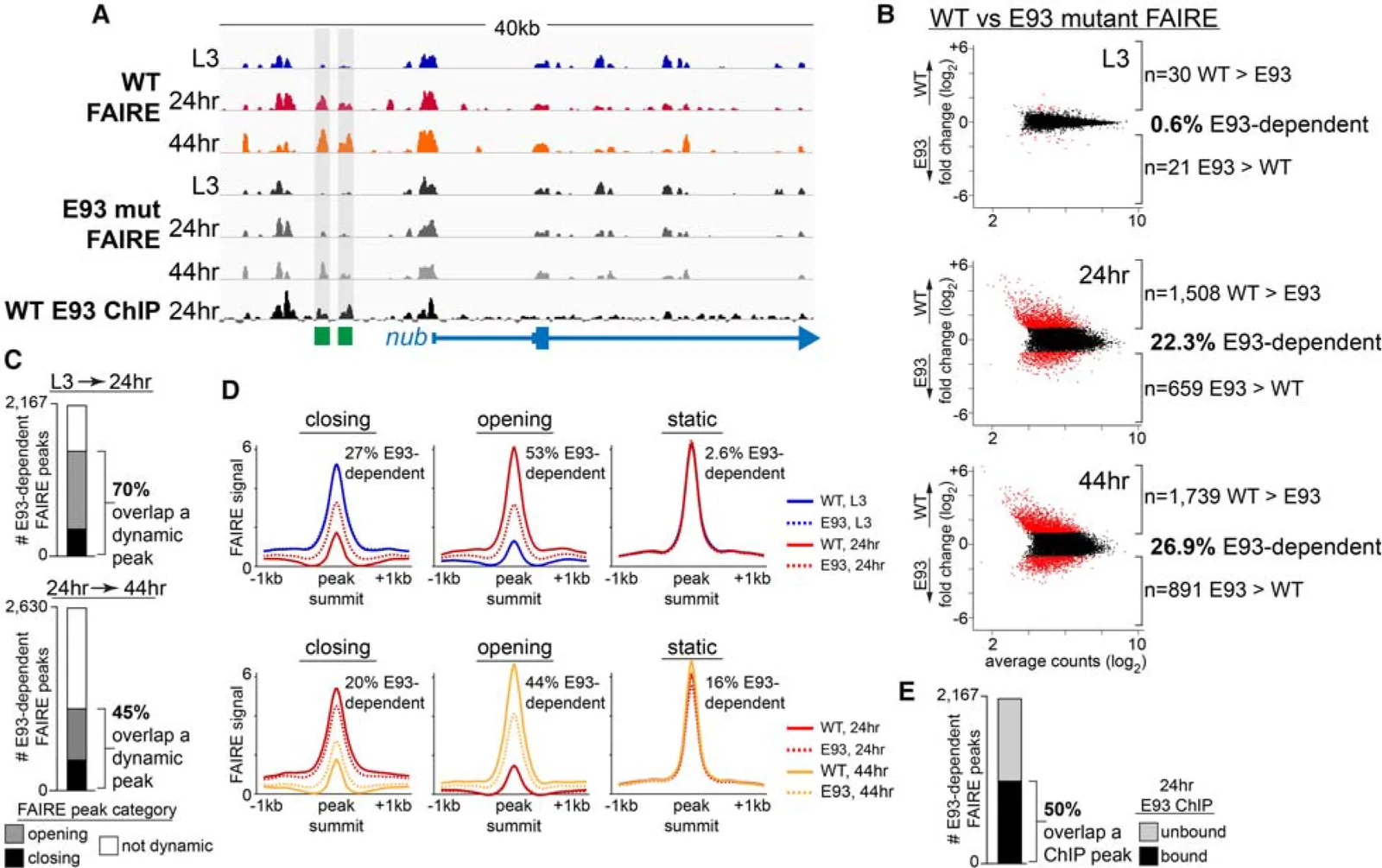
E93 binding is required for temporally dynamic open chromatin changes. (*A*) Browser shot showing FAIRE-seq signal from wild-type and *E93* mutant wings. E93 ChIP-seq signal from wild-type 24-h wings is shown in black. The *nub*^*vein*^ and *nub*^*margin*^ enhancers are shown in green. (*B*) MA plots of FAIRE-seq signal in the top 7699 FAIRE peaks from each wild-type and *E93* mutant wing data set. Differentially accessible peaks are colored red. (*C* ) Stacked bar plots of the fraction of E93-dependent FAIRE peaks that overlap a temporally dynamic FAIRE peak. (*D*) Line plots of the average FAIRE-seq signal in FAIRE peaks that close, open, or remain unchanged between consecutive time points. The percentage of FAIRE peaks in each category that are E93-dependent is shown. Solid lines show wild-type FAIRE-seq signal. Dashed lines show *E93* mutant FAIRE-seq signal. (*E*) Stacked bar plot showing the fraction of E93-dependent FAIRE peaks that overlap an E93 ChIP peak.

The high degree of overlap between E93 binding and temporally dynamic open chromatin sites suggests that E93 may play a direct role in controlling chromatin accessibility during pupal wing development. To test this hypothesis, we performed FAIRE-seq in *E93* mutant wings at L3, 24 h, and 44 h (Fig. 6A). In L3 wings, we observed very few changes in open chromatin between wild-type and *E93* mutants (Fig. 6B), consistent with expectations, since E93 is not yet expressed at this time. In contrast, we observed thousands of changes in open chromatin between wild-type and *E93* mutant wings at 24 h and 44 h. For example, 1508 FAIRE peaks out of the top 7699 peaks from each pair of data sets are more open in wild-type wings than in *E93* mutants at 24 h (Fig. 6B), demonstrating that E93 is required for accessibility at these sites. Surprisingly, 659 FAIRE peaks are more open in *E93* mutant wings than in wild type at 24 h, indicating that E93 is not required to promote accessibility at these sites; instead, it is required for the opposite: promoting nucleosome occupancy. Thus, loss of E93 results in not only the loss of accessibility at thousands of sites in the genome but also the inappropriate presence of accessible chromatin at hundreds of additional sites.

We next asked whether sites that depend on E93 for proper chromatin accessibility correspond to temporally dynamic FAIRE peaks. Indeed, 70% of E93-dependent FAIRE peaks are temporally dynamic between L3 and 24 h in wild-type wings (Fig. 6C). This includes 53% of sites that normally open between L3 and 24 h in wild-type wings but fail to open in *E93* mutants and 27% of sites that normally close between L3 and 24 h in wild-type wings but fail to close in *E93* mutants (Fig. 6D; Supplemental Fig. S7A). In contrast, only 4% of sites that change in accessibility during embryogenesis overlap an E93-dependent FAIRE peak (Fisher's exact test *P*-value <2.2 × 10^−16^) (Supplemental Fig. S7C). FAIRE peaks that do not change in accessibility between L3 and 24 h in wild-type wings exhibit no change in accessibility in *E93* mutants (Fig. 6D, “static” peaks). We obtained similar results for the 24- to 44-h interval. The lower number of E93-dependent FAIRE peaks that overlap a dynamic FAIRE peak at this later time interval (Fig. 6C; Supplemental Fig. S7D) is possibly due to a persistent failure in chromatin accessibility from the earlier time point, such as sites that fail to open in *E93* mutants at 24 h and stay closed at 44 h (e.g., highlighted region in Fig. 6A). Similarly, these indirect effects likely explain the increase in the fraction of static peaks that exhibit E93 dependence during this time interval (Fig. 6D). Importantly, we found that 50% of FAIRE peaks that are dependent on E93 for accessibility at 24 h are directly bound by E93 (Fig. 6E). Together, these findings demonstrate that E93 controls temporal progression of development by directly and indirectly regulating the accessibility of thousands of sites in the genome. In the absence of E93, nearly half of the expected open chromatin changes fail to occur. One consequence of this failure is that *E93* mutants exhibit a heterochronic open chromatin defect: Open chromatin profiles of *E93* mutant wings at 24 h are as similar to those of wild-type wings at L3 as they are to those of wild-type wings at 24 h (Supple-mental Fig. S8).

### E93 controls temporal-specific enhancer activity through three distinct mechanisms

**Figure 7.**
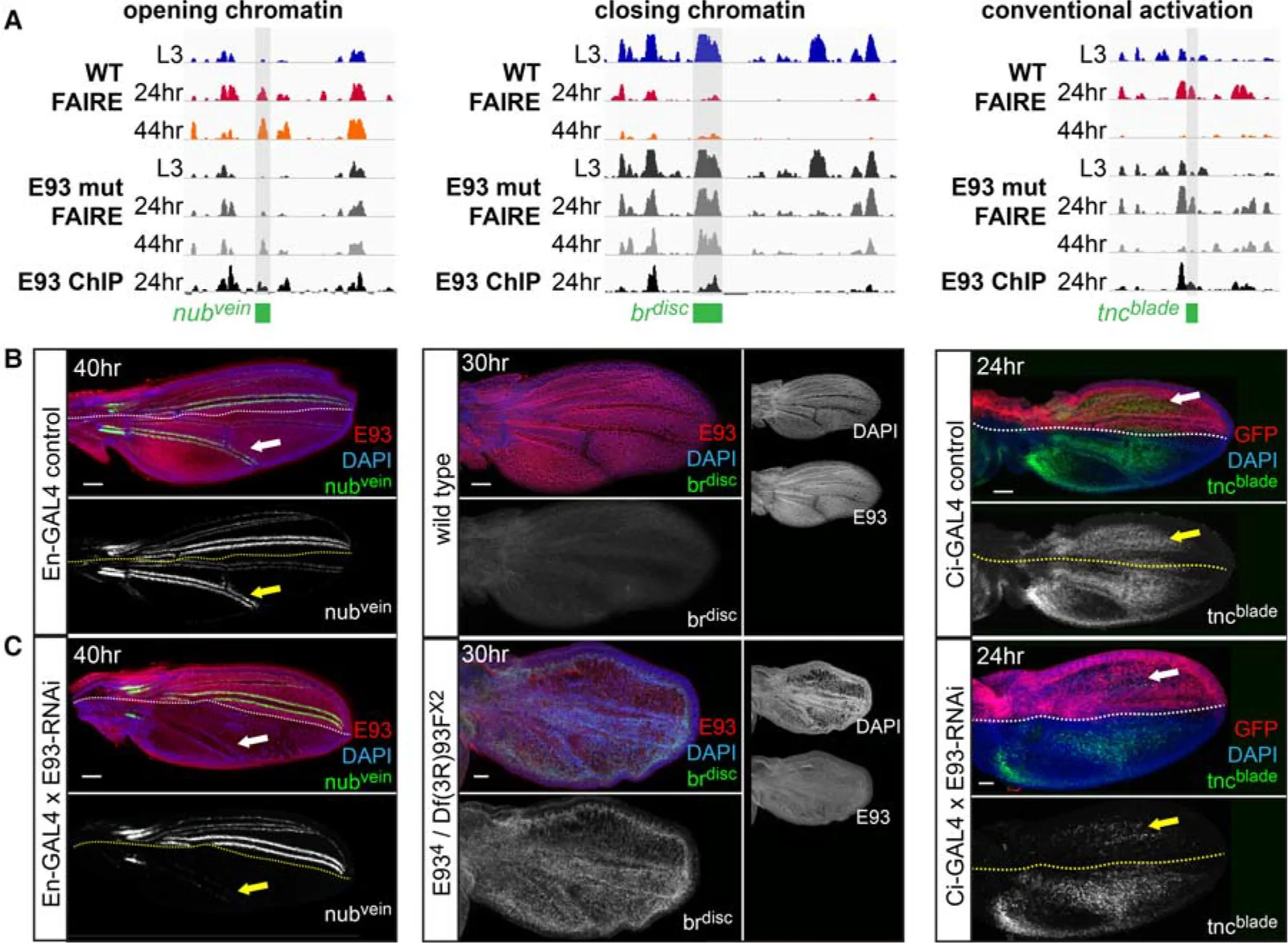
E93 controls temporal-specific enhancer activity through three distinct modalities. (*A*) Browser shots of FAIRE-seq and ChIP-seq signal from wild-type and *E93* mutant wings at the indicated loci. (*B*,*C* ) Immunostaining of reporter activity for each indicated enhancer. (*B*) Reporter activity in control or wild-type wings. (*C*) Reporter activity (green) in wings expressing E93 RNAi under control of *Ci-GAL4* (*tnc*^*blade*^) or *En-GAL4* (*nub*^*vein*^) or in *E93* mutant wings (*br*^*disc*^). The dotted lines indicate the boundary between RNAi-expressing and RNAi-nonexpressing cells. Arrows indicate loss of reporter activity. Bars, 50 µm.

The results described above suggest that the developmental defects observed in *E93* mutants are due to a failure to make temporally required changes in the accessibility of DNA regulatory elements genome-wide. To directly test this hypothesis, we examined the consequences of E93 loss-of-function on the activity of the temporally dynamic transcriptional enhancers that we identified above. ChIP-seq shows that the *nub*^*vein*^ enhancer is directly bound by E93 at 24 h, and FAIRE-seq in wild-type wings shows that *nub*^*vein*^ progressively opens after the L3 stage (Fig. 7). The timing of this accessibility coincides with increasing *nub*^*vein*^ enhancer activity in wing veins (Fig. 4). FAIRE-seq from *E93* mutant wings reveals that this enhancer is dependent on E93 for its accessibility: It fails to open at 24 h and remains closed at 44 h in the absence of E93 (Fig. 7). Using the GAL4-UAS system to drive an E93 RNAi construct specifically in the posterior compartment of the wing with *En-GAL4*, we observed a strong loss of *nub*^*vein*^ activity upon E93 knockdown specifically in the regions where the RNAi was expressed (Fig. 7). The enhancer remains active in only a few cells in the proximal wing after E93 knockdown, and most RNAi-expressing cells show complete loss of GFP. Thus, the failure to open the *nub*^*vein*^ enhancer in *E93* mutant flies correlates with a failure to activate the enhancer in transgenic reporter assays.

We next examined the *br*^*disc*^ enhancer, which is open and active in L3 wings but closed and inactive in 24-h and 44-h wings (Fig. 3). ChIP-seq reveals that E93 is directly bound to this enhancer, and FAIRE-seq shows that it remains persistently open at 24 h and 44 h in *E93* mutant wings (Fig. 7). Consistent with this persistent accessibility, *br*^*disc*^ is expressed in *E93* mutants at 24 h, when it would normally be off in wild-type wings (Fig. 7). The *br*^*disc*^ pattern in 24-h E93 mutant wings is nearly ubiquitous, similar to its pattern earlier in L3 wings. Thus, E93 is required to close this enhancer after the L3 stage, and failure to do so results in its aberrant expression at later developmental stages.

Finally, we examined the *tnc*^*blade*^ enhancer, which is open and active in 24-h wings (Fig. 3). ChIP-seq shows that E93 is directly bound to *tnc*^*blade*^ (Fig. 7). However, despite this binding, *tnc*^*blade*^ does not significantly change in accessibility in *E93* mutant wings, demonstrating that E93 is not required for promoting the accessibility of this enhancer. Nevertheless, RNAi-mediated knockdown of E93 in the anterior compartment of the wing using *Ci-GAL4* results in loss of *tnc*^*blade*^ activity in RNAi-expressing cells. Thus, while the *tnc*^*blade*^ enhancer is not dependent on E93 for its accessibility, it is still dependent on E93 for its activity. Importantly, the mutual dependence of the *nub*^*vein*^ and *tnc*^*blade*^ enhancers on E93 for transcriptional activity, combined with the specific dependence of *nub*^*vein*^ on E93 for accessibility, suggests that distinct bio-chemical mechanisms underlie E93 function at these enhancers.

## Discussion

The mechanisms controlling transcription factor targeting in the genome are incompletely understood, particularly in the context of animal development. Here, we show that the hormone-induced transcription factor E93 plays a direct role in controlling temporal changes in chromatin accessibility in the developing *Drosophila* wing. Together with our previous findings, this work supports a model in which two axes of information regulate enhancer activity in developing appendages: Temporal information is provided by hormone-induced transcription factors that regulate accessibility of transcriptional enhancers, and spatial information is provided by the appendage master transcription factors that differentially regulate the activity of these enhancers.

### Transcription factor targeting and temporal gene regulation

The importance of master transcription factors in specifying spatial identity during development suggests that they may control where other transcription factors bind in the genome. One prediction of this model is that tissues whose identities are determined by different master transcription factors would exhibit different genome-wide DNA-binding profiles. However, we found recently that the *Drosophila* appendages (wings, legs, and halteres), which use different transcription factors to determine their identities, share nearly identical open chromatin profiles. Moreover, these shared open chromatin profiles change coordinately over developmental time. There are two possible explanations for these findings. Either (1) different transcription factors produce the same open chromatin profiles in different appendages or (2) transcription factors shared by each appendage control open chromatin profiles instead of the master transcription factors of appendage identity. We favor the second model for several reasons. Since the appendage master transcription factors possess different DNA-binding domains with distinct DNA-binding specificities, it is unlikely for them to bind the same sites in the genome. Supporting this expectation, ChIP for Scalloped and Homothorax, two transcription factors important for appendage identity, shows clear tissue-specific binding in both the wing and eye–antennal imaginal discs (Slattery et al. 2013). We also prefer the second model because it provides a relatively straightforward mechanism for the observed temporal changes in open chromatin: By changing the expression of the shared temporal transcription factor over time, the open chromatin profiles that it controls would change as well. In contrast, expression of appendage master transcription factors is relatively stable over time, making it unlikely for them to be sufficient for temporal changes in open chromatin.

We propose that control of chromatin accessibility in the appendages is mediated at least in part by transcription factors downstream from ecdysone signaling. According to this model, a systemic pulse of ecdysone initiates a temporal cascade of hormone-induced transcription factor expression in each of the appendages. We thus refer to these as “temporal” transcription factors. Temporal transcription factors can directly regulate the accessibility of transcriptional enhancers by opening or closing them, thereby conferring temporal specificity to their activity and driving development forward in time. Master transcription factors then bind accessible enhancers depending on their DNA-binding preferences (or other means of binding DNA) and differentially regulate the activity of these enhancers to control spatial patterns of gene expression, thus shaping the unique identities of individual appendages.

Our experiments with E93 provide direct support for this model. In wild-type wings, thousands of changes in open chromatin occur after the large pulse of ecdysone that triggers the end of larval development. In *E93* mutants, ∼40% of these open chromatin changes fail to occur. Importantly, nearly three-quarters of sites that depend on E93 for accessibility correspond to temporally dynamic sites in wild-type wings. Thus, chromatin accessibility is not grossly defective across the genome; instead, defects occur specifically in sites that change in accessibility over time. This finding, combined with the large fraction of temporally dynamic sites that depend on E93 for accessibility, indicates that E93 controls a genome-wide shift in the availability of temporal-specific transcriptional enhancers. Supporting this hypothesis, we show that temporal-specific enhancers depend on E93 for both accessibility and activity. Since we propose that the response to ecdysone is shared across the appendages, we predict that similar defects occur in appendages besides the wing. It remains to be seen whether other ecdysone-induced transcription factors besides E93 control accessibility of enhancers at different developmental times. It also remains to be seen how the temporal transcription factors work with the appendage master transcription factors to control appendage-specific enhancer activity.

### Mechanisms of temporal transcription factor function

Our findings suggest that E93 controls temporal-specific gene expression through three different modalities that potentially rely on three distinct biochemical activities. The enrichment of E93 motifs and binding of E93 to temporally dynamic sites indicate that it contributes to this regulation directly. We propose that these combined activities drive development forward in time by turning off early-acting enhancers and simultaneously turning on late-acting enhancers.

First, as in the case of the *tnc*^*blade*^ enhancer, E93 appears to function as a conventional activator. In the absence of E93, *tnc*^*blade*^ fails to express at high levels, but the accessibility of the enhancer does not measurably change. This suggests that binding of E93 to *tnc*^*blade*^ is required to recruit an essential coactivator. Importantly, this finding demonstrates that E93 is not solely a regulator of chromatin accessibility. E93 binds many open chromatin sites in the genome without regulating their accessibility and thus may regulate the temporal-specific activity of many other enhancers. In addition, since the *tnc*^*blade*^ enhancer opens between L3 and 24 h even in the absence of E93 (Fig. 7A), there must be other factors that control its accessibility, perhaps, for example, transcription factors induced by ecdysone earlier in the temporal cascade.

Second, as in the case of the *nub*^*vein*^ enhancer, E93 is required to promote chromatin accessibility. In this capacity, E93 may function as a pioneer transcription factor to open previously inaccessible chromatin. Alternatively, E93 may combine with other transcription factors, such as the wing master transcription factors, to compete nucleosomes off DNA. Testing the ability of E93 to bind nucleosomal DNA will help to discriminate between these two alternatives. In either case, we propose that this function of E93 is necessary to activate late-acting enhancers across the genome. Since only half of E93-dependent enhancers are directly bound by E93 at 24 h (Fig. 6E), it is also possible that E93 regulates the expression of other transcription factors that control chromatin accessibility. Alternatively, if E93 uses a “hit and run” mechanism to open these enhancers, our ChIP time point may have been too late to capture E93 binding at these sites.

Finally, as in the case of the *br*^*disc*^ enhancer, E93 is required to decrease chromatin accessibility. We propose that this function of E93 is necessary to inactivate early-acting enhancers across the genome. Current models of gene regulation do not adequately explain how sites of open chromatin are rendered inaccessible, but the ability to turn off early-acting enhancers is clearly an important requirement in developmental gene regulation. It may also be an important contributor to diseases such as cancer, which exhibits widespread changes in chromatin accessibility relative to matched normal cells (Stergachis et al. 2013). Thus, this role of E93 may represent a new functional class of transcription factor (“reverse pioneer”) or conventional transcriptional repressor activity. Additional work is required to decipher the underlying mechanisms. Notably, recent work on the temporal dynamics of iPS cell reprogramming suggest a similar role for Oct4, Sox2, and Klf4 in closing open chromatin to inactivate somatic enhancers (Chronis et al. 2017).

## Materials and methods

### Drosophila *culture and genetics*

Flies were grown at 25°C under standard culture conditions. The genotype of the wild-type strain was w^1118^/yw, hs-FLP. Late wandering third instar larvae were used for the L3 stage. White prepupae were used as the 0-h time point for pupal staging. The following genotypes were also used: *UAS-E93 RNAi* (Vienna *Drosophila* Resource Center, no. 104390), *E93 protein trap* (*Drosophila* Gene Disruption Project, Bloomington *Drosophila* Stock Center [BDSC], no. 43675), *UAS-nls-GFP* (chromosome 2; BDSC, no. 4775), *UAS-nls-GFP* (chromosome 3; BDSC, no. 4776), *E93*^*4*^ (gift of Craig Woodard), *Df(3R)93F*^*x2*^ (gift of Eric Baehrecke), and *UAS-destabilized-GFP* (gift of Brian McCabe).

### Sample preparation for high-throughput sequencing

A minimum of 40 wings were dissected from staged female flies in 1 × PBS and transferred to ice for subsequent processing. RNA was prepared as described previously (Guo et al. 2016), and the KAPA stranded mRNA-seq kit was used for library construction. FAIRE-seq was performed as described previously (McKay and Lieb 2013), and the Rubicon Thruplex DNA-seq kit was used for library construction. ChIP-seq was performed on 24-h ± 1-h manually dissected wings (*n* = 717 Rep1; *n* = 1280 Rep2) from both male and female E93 protein trap flies. Wings were dissected in 1 × PBS and kept on ice. Batches of wings from 20 pupae were fixed in 4% paraformaldehyde, 50 mM HEPES, 100 mM NaCl, 1 mM EDTA, and 0.5 mM EGTA for 20 min at room temperature followed by quenching with 125 mM glycine in 1 × PBS and 0.01% Triton. Fixed wings were dounce-homogenized in 10 mM HEPES, 10 mM EDTA, 0.5 mM EGTA, 0.25% Triton, and 1 mM PMSF. Nuclei were pelleted at 4500*g* for 20 min and resuspended in 10 mM HEPES, 200 mM NaCl, 1 mM EDTA, 0.5 mM EGTA, 0.01% Triton, and 1 mM PMSF. After nutating for 10 min at 4° C, nuclei were pelleted again and resuspended in 140 mM NaCl, 10 mM HEPES, 1 mM EDTA, 0.5 mM EGTA, 1 mM PMSF, and 0.1% SDS followed by sonication on ice with a Branson sonifier until the average chromatin fragment size was 200 base pairs. The soluble chromatin fraction was used for ChIP. Briefly, extracts were precleared with protein A Dynabeads for 2 h at 4°C, and cleared extracts were incubated with 5 µg of rabbit anti-GFP antibody (Abcam, ab290) overnight at 4°C. Bead pull-down was performed for 3 h the following day. Antibody–bead complexes were washed successively and then eluted with 1% SDS, 250 mM NaCl, 10 mM Tris, and 1 mM EDTA. Samples were treated with RNase A and proteinase K and heated overnight at 65°C to reverse cross-links, and purified DNA was recovered by phenol-chloroform/ethanol precipitation. Rubicon Thruplex DNA-seq kit was used for library construction. All samples were sequenced on an Illumina HiSeq 2500 at the University of North Carolina High-Throughput Sequencing Facility. Addition-al details are available on request.

### Sequencing data analysis

Sequencing reads were aligned to the dm3 reference genome. RNA-seq analysis was performed as described previously (Guo et al. 2016). We defined differentially expressed genes as those having a logCPM >2 in at least one sample and changing by at least twofold between pairwise time points. GO analysis was performed using DAVID (Database for Annotation, Visualization, and Integrated Discovery) (Huang da et al. 2009) and REViGO (reduce and visualize GO) (Supek et al. 2011). FAIRE-seq analysis and peak calling were performed as described previously (McKay and Lieb 2013; Schulz et al. 2015). FAIRE-seq and ChIP-seq data were visualized using Integrative Genomics Viewer (Robinson et al. 2011). *Z*-scores were calculated using the mean and standard deviation per chromosome arm. To focus on a high-confidence set of peaks, we chose a MACS2 −log_10_-adjusted *P*-value of 40 from the 44-h wild-type data set and selected an equivalent number of peaks (*n* = 7699) from the remaining data sets. edgeR (Robinson et al. 2010) was used for differential peak calling, as described previously (Schulz et al. 2015). Briefly, BedTools (Quinlan and Hall 2010) was used to calculate read depth for each set of peaks. FAIRE peaks with an FDR <0.05 that changed greater than twofold were defined as differentially accessible. We defined E93-dependent peaks as those called as differentially accessible (open or closed) in E93 mutant wings relative to wild type. Heat maps were generated using deepTools version 2.4.0 (Ramirez et al. 2014). Average signal line plots were generated from *Z*-normalized data using the Bioconductor packages rtracklayer version 1.32.2 (Lawrence et al. 2009), GenomicRanges version 1.24.3 (Lawrence et al. 2013), and Genomation version 1.4.2 (Akalin et al. 2015) along with custom R scripts (available on request). DNA-binding motifs used for enrichment analysis were obtained from FlyFactorSurvey (Zhu et al. 2011), and enrichment was measured using the AME tool (McLeay and Bailey 2010) in MEME (Bailey et al. 2015) by comparing temporally dynamic peaks in each category with static peaks (defined as those changing <1.3-fold between consecutive time points). Only motifs with an adjusted *P*-value <0.05 were plotted, and only the lowest *P*-value was reported for each transcription factor to remove redundancy. Data are available from Gene Expression Omnibus under the accession number GSE97956.

### Transgenic reporter analysis and immunofluorescence

Candidate enhancers were cloned from wild-type *y; cn, bw, sp* genomic DNA based solely on open chromatin data. Gateway (Invitrogen) cloning was used to move candidate enhancers into destination vectors. Injections were performed at BestGene. The *br*^*disc*^ and *br*^*ade*^ enhancers were cloned into the pφUGG GAL-4 destination vector (Jiang et al. 2010) and integrated into the *attP40* site on chromosome 2; this vector was chosen to allow different reporters to be driven by GAL4 (e.g., UAS-destabilized GFP). The *nub*^*vein*^ and *nub*^*margin*^ enhancers were cloned into a modified pDEST-HemmarG vector (Han et al. 2011) in which the CD4 transmembrane domain was replaced with an SV40 nuclear localization signal (PKKKRKV). The *tnc*^*wv*^ and *tnc*^*blade*^ enhancers were cloned into a modified pDEST-HemmarR vector (Han et al. 2011) in which the CD4 transmembrane domain was replaced with the SV40 nuclear localization signal; this tdTomato vector was chosen to allow the combining of the *tnc* reporters with existing GFP-marked GAL4 drivers. Each *nub* and *tnc* enhancer was integrated into the *attP2* site on chromosome 3. Integration of each reporter into its respective *attP* site was confirmed by PCR. Immunostaining and confocal imaging were performed as described previously (McKay and Lieb 2013). The following antibodies were used: rabbit anti-GFP (1:1000; Abcam, ab290), mouse anti-phospho-tyrosine (1:1000; Fisher Scientific, clone 4G10), and rabbit anti-E93 (1:2500; this study). Polyclonal antibodies to E93 were raised in rabbits using amino acid sequences 271–520 of the E93-PA isoform, which is present in all annotated E93 isoforms. In some cases, 30-h wings were used in figure images due to their ease of mounting relative to 24-h wings. In all cases, reporter analysis was also conducted at 24 h, and no significant differences in reporter pattern were observed between 24 h and 30 h. All primer sequences and vectors are available on request.
