# Supplementary material for: Hormone-dependent control of developmental timing through regulation of chromatin accessibility
Source: Genes Dev. 2017 May 1;31(9):862–75. doi: 10.1101/gad.298182.117 (PMC5458754; doi:10.1101/gad.298182.117)
Supplement: Supplemental Material [file supp_31_9_862__index.html]

Hormone-dependent control of developmental timing through regulation of chromatin accessibility — Supplemental Material 

# Hormone-dependent control of developmental timing through regulation of chromatin accessibility

## Supplemental Material

- Supplemental\_Figure\_Legends.pdf
- Supplemental\_TableS2.xlsx
- Supplemental\_Figures.pdf
- Supplemental\_TableS3.txt
- Supplemental\_TableS1.xlsx
