# Supplementary material for: Hormone-dependent control of developmental timing through regulation of chromatin accessibility
Source: Genes Dev. 2017 May 1;31(9):862–75. doi: 10.1101/gad.298182.117 (PMC5458754; doi:10.1101/gad.298182.117)
Supplement: Supplemental Material [file supp_gad.298182.117_Supplemental_Figure_Legends.pdf]

Supplemental Figure Legends

Supplemental Figure S1. Gene ontology analysis of pupal wing RNA-seq data. Bar plots of the p value for GO term enrichment (Biological Process) for differentially expressed genes between consecutive time points.

Supplemental Figure S2. FAIRE-seq profiles show temporally dynamic open chromatin in pupal wings. (**A**) Stacked bar plots showing overlap between gene promoters and FAIRE peaks at fixed distances away. Genes that increase in expression are more likely to overlap a nearby FAIRE peak that is opening between the time intervals. Conversely, genes that decrease in expression are more likely to overlap a nearby FAIRE peak that is closing between 24hr to 44hr. However, genes that decrease in expression between L3 and 24hr are not more likely to overlap a nearby FAIRE peak that is closing. It is not clear why this happens. (**B**) MA plots showing FAIRE-seq signal in the union set of FAIRE peaks for consecutive time points in wing development. Differentially accessible peaks are colored red (edgeR FDR < 0.05, fold change > 2). (**C**) Stacked bar plots showing the fraction of FAIRE peaks that change between consecutive time points. (**D**) Pie charts showing the overlap of dynamic FAIRE peaks with proximal promoter sequences (+/- 500bp transcription start sites) for each category of dynamic FAIRE peak.

Supplemental Figure S3. Additional time points for the enhancers depicted in Figure 3. (**top row**) Browser shots of FAIRE-seq signal from the *tnc* (**A**), *nub* (**B**), and *br* (**C**) loci. (**remaining rows**) Confocal images of wings at time points approximately coinciding with the FAIRE-seq time course. Persistent tnc^{blade} reporter activity at 44hr (**A**) is likely a consequence of perdurance of tdTomato protein (green), since there are no cell divisions between 24hr and 44hr in pupal wings.

Supplemental Figure S4. Additional examples of temporally dynamic open chromatin sites corresponding to temporal specific enhancers. (**top row**) Browser shots of FAIRE-seq signal from the *tnc* (**A**), *nub* (**B**), and *br* (**C**) loci. (**remaining rows**) Confocal images of wings at early and late time points for the indicated enhancers. The absence of br^{ade} reporter activity in 30hr wings (**C**) may be due to the absence of adepithelial cells in the wing blade at this stage of development.

Supplemental Figure S5. The E93 protein trap recapitulates E93 expression in pupal wings. (**top**) Immunostaining of three stages of wing development. DAPI in blue, GFP in green, E93 antibodies in red. White squares indicate zoomed regions. (**bottom**) Table indicating the number of progeny from a cross between flies bearing the $E93^{GFSTF}$ protein trap chromosome and flies bearing a deficiency that deletes the *E93* locus. Because E93 loss of function mutants are recessive lethal, the viability of $E93^{GFSTF}/Df(3R)93F^{X2}$ progeny indicates that the E93 protein trap is functional.

Supplemental Figure S6. E93 ChIP-seq signal is correlated with, but distinct from, 24hr FAIRE-seq signal. (**A**) Scatterplot of E93 ChIP-seq and 24hr FAIRE-seq signals for all 24hr FAIRE peaks. FAIRE peaks that overlap an E93 ChIP peak (>50%) are colored red. (**B**) Pie charts showing overlap of E93 ChIP-seq and 24hr FAIRE-seq peaks with transcription start sites of annotated genes (+/- 500bp).

Supplemental Figure S7. E93-dependent peaks exhibit temporally-dynamic changes in wild type wings. (**A**) Line plots of the average FAIRE-seq signal in FAIRE peaks that close, open, or remain unchanged between L3 and 24hr, separated into E93-bound and E93-unbound categories, as determined by E93 ChIP-seq. (**B**) Same as in **A**, but with 24hr to 44hr data. (**C, D**) Stacked bar plot of the fraction of temporally dynamic FAIRE peaks between L3 and 24hr (**C**) and between 24hr and 44hr (**D**) that overlap an

E93-dependent FAIRE peak, as compared to the fraction of temporally dynamic FAIRE peaks across two stages of embryogenesis (e2-4hr to e16-18hr) (p values from Fisher's exact test).

Supplemental Figure S8. E93 mutant wings show heterochronic open chromatin defects. Heat map of Pearson correlation coefficients for each wild type and E93 mutant FAIRE-seq replicate. Note the increased similarity of E93 mutant open chromatin profiles relative to wild type open chromatin profiles of earlier developmental stages, indicating that the failure to change over time results in chronologically later mutant wings resembling those of an earlier developmental stage.
