# Supplementary material for: Hormone-dependent control of developmental timing through regulation of chromatin accessibility
Source: Genes Dev. 2017 May 1;31(9):862–75. doi: 10.1101/gad.298182.117 (PMC5458754; doi:10.1101/gad.298182.117)

GO Term (Biological Process) for differentially expressed genes in wings

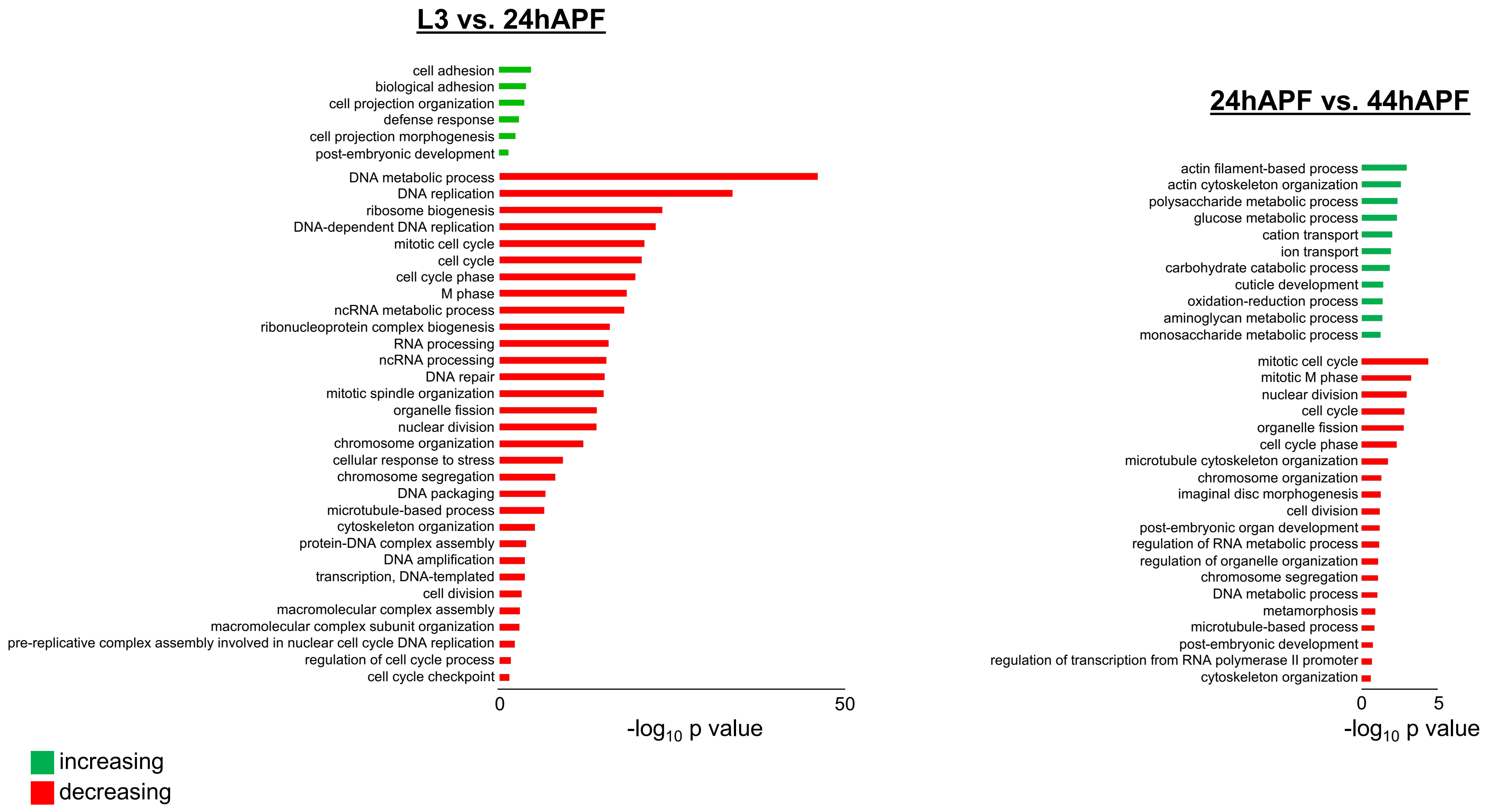

Open chromatin profiles are temporally dynamic in pupal wings

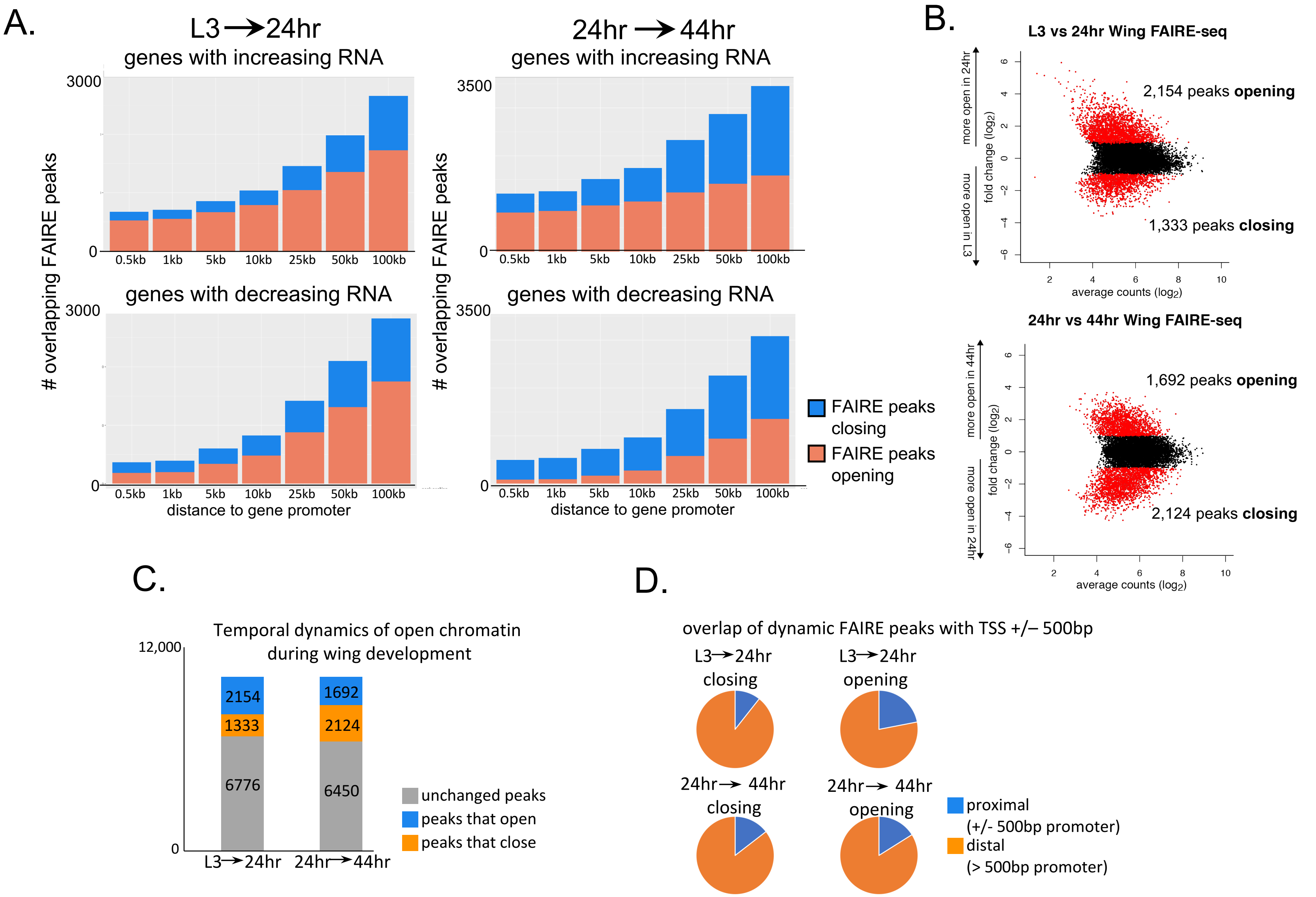

Uyehara_Supplemental_FigS3

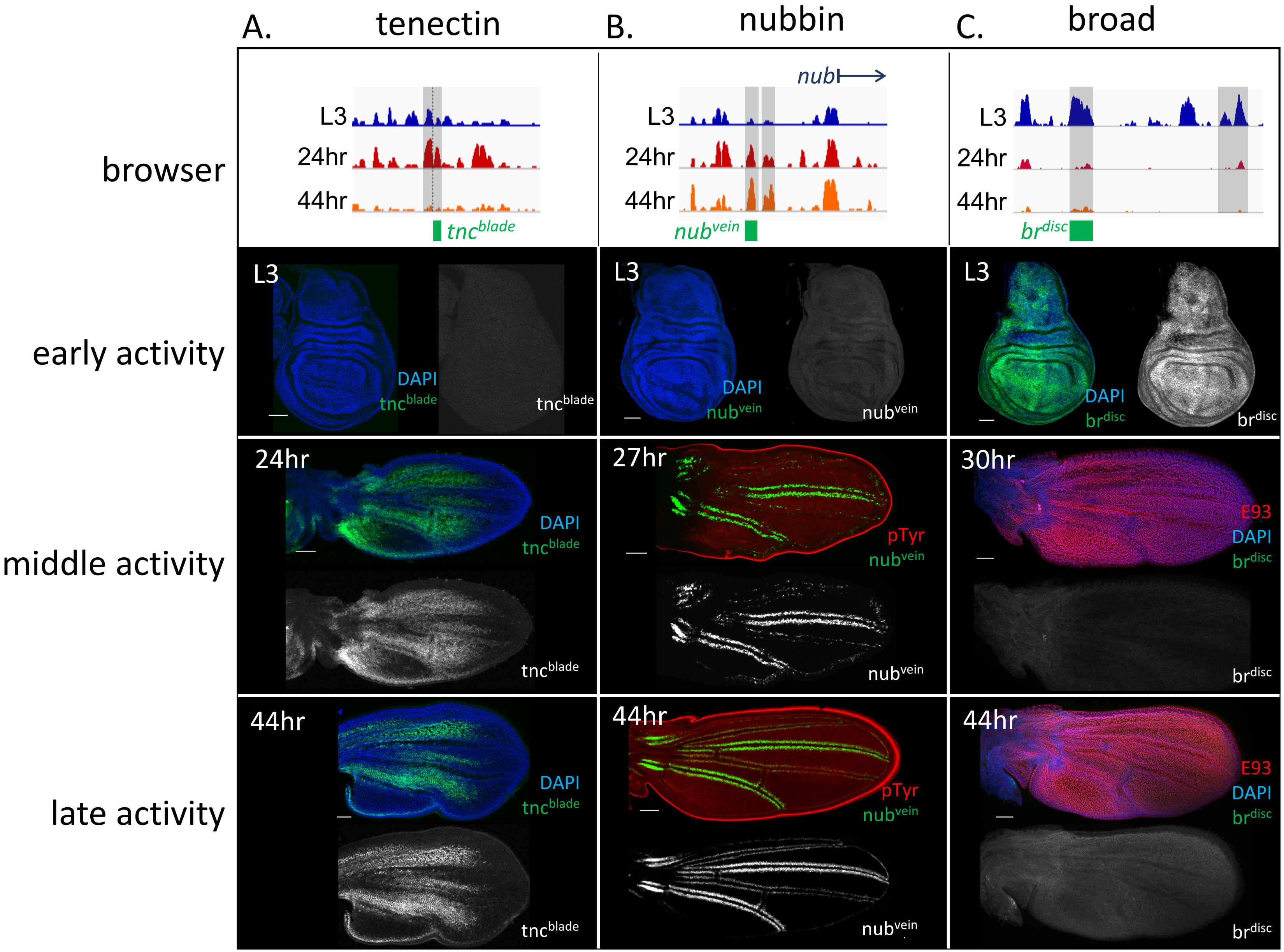

Uyehara_Supplemental_FigS4

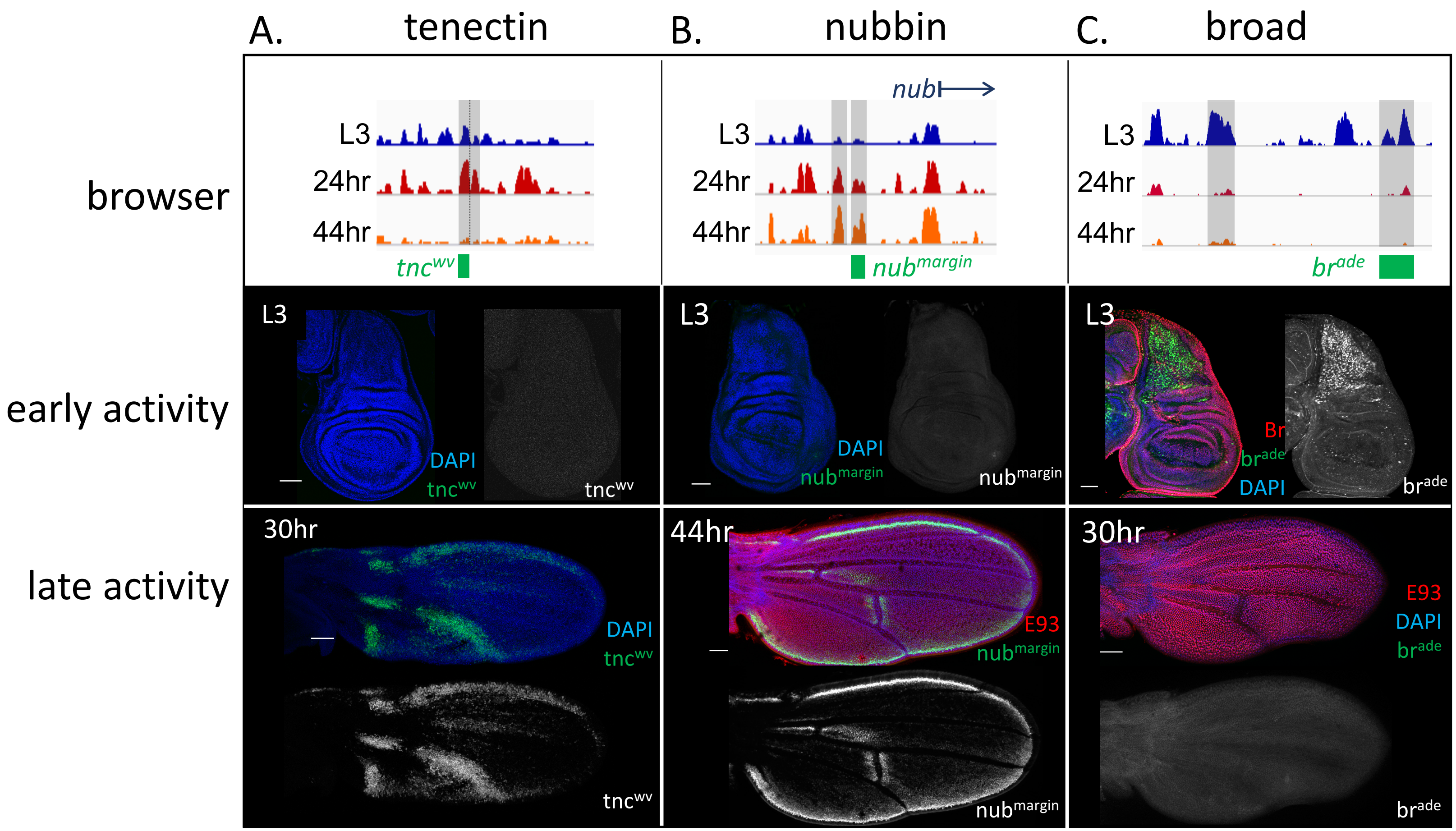

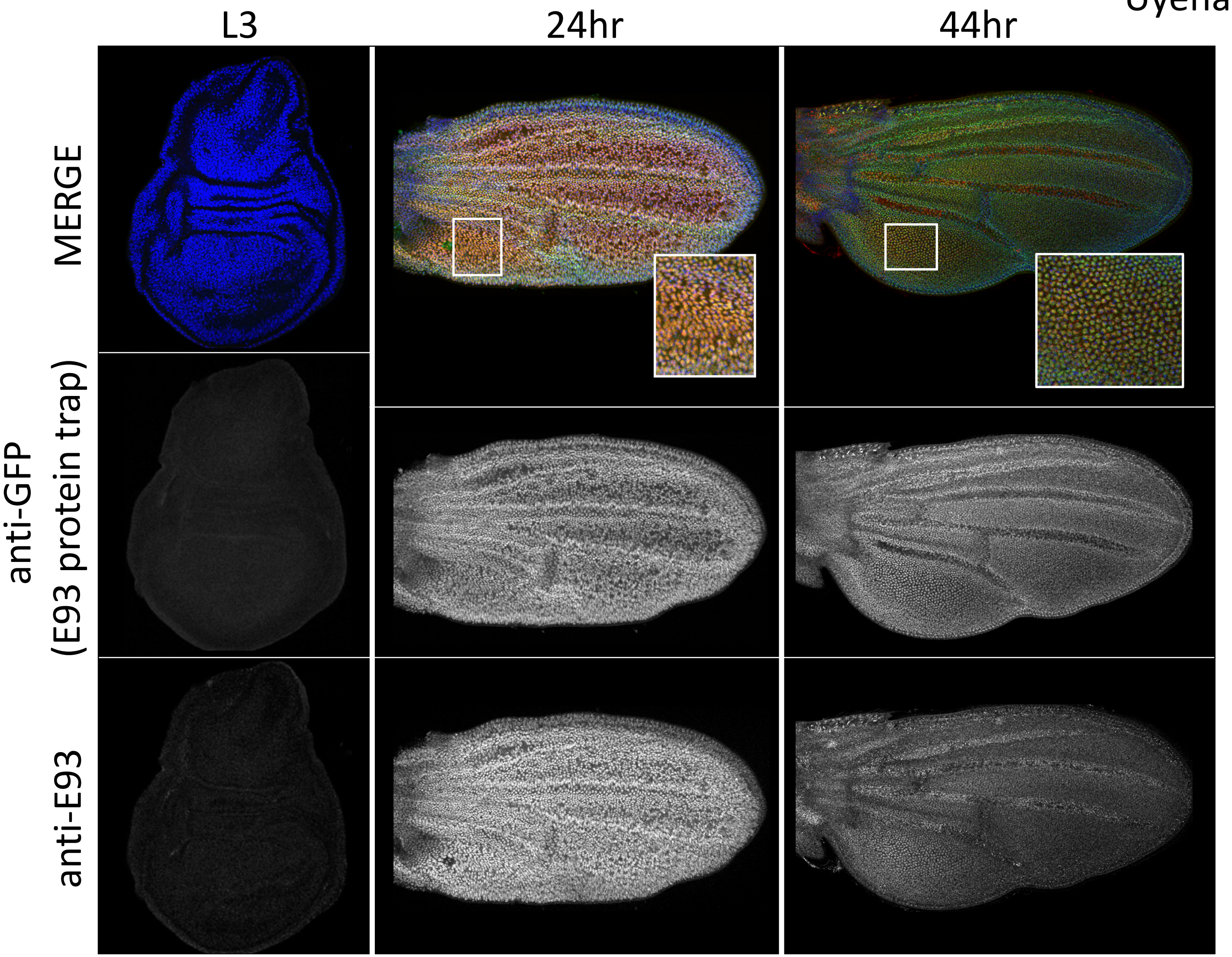

cross:

$$\frac{E93^{GFSTF}}{TM3} \times \frac{Df(3R)93F^{X2}}{TM6B}$$

# progeny:	*Df(3R)93F^{X2}*	*TM6B, tb*
E93^{GFSTF}	212	265
TM3, sb	251	157

Uyehara_Supplemental_FigS6

Scatter plot of FAIRE vs E93 ChIP signal in 24hr FAIRE peaks

A.

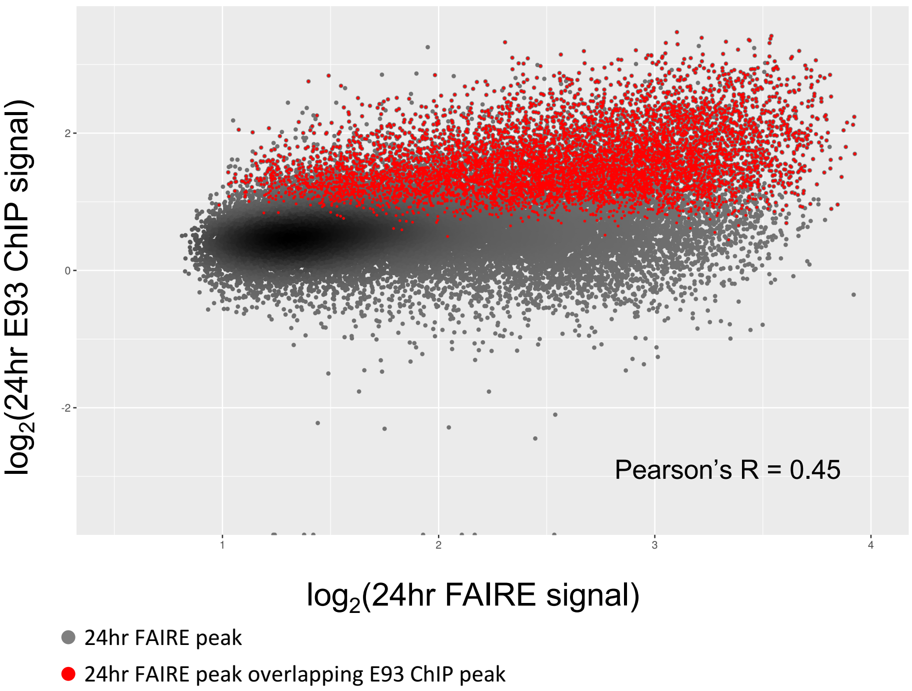

B.

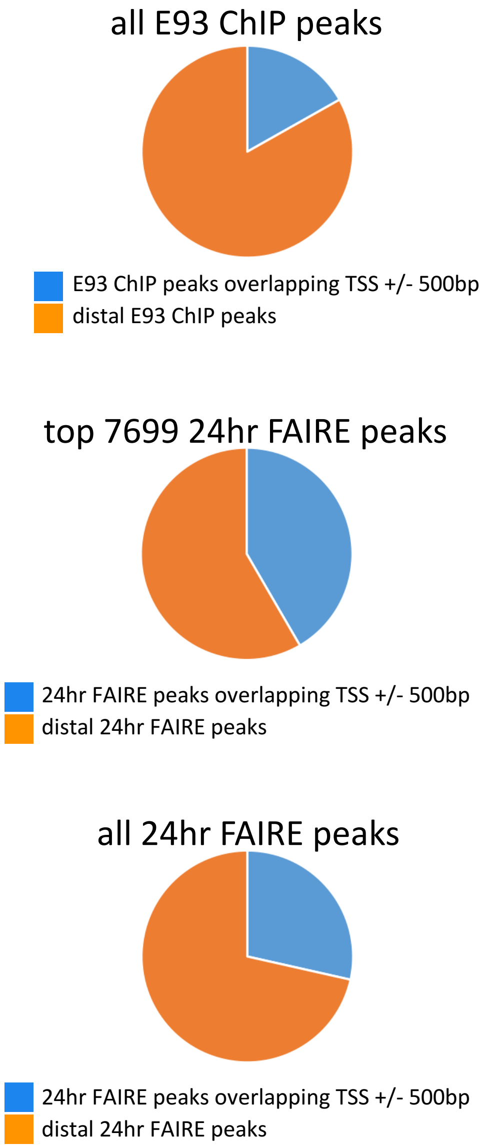

Uyehara_Supplemental_FigS7

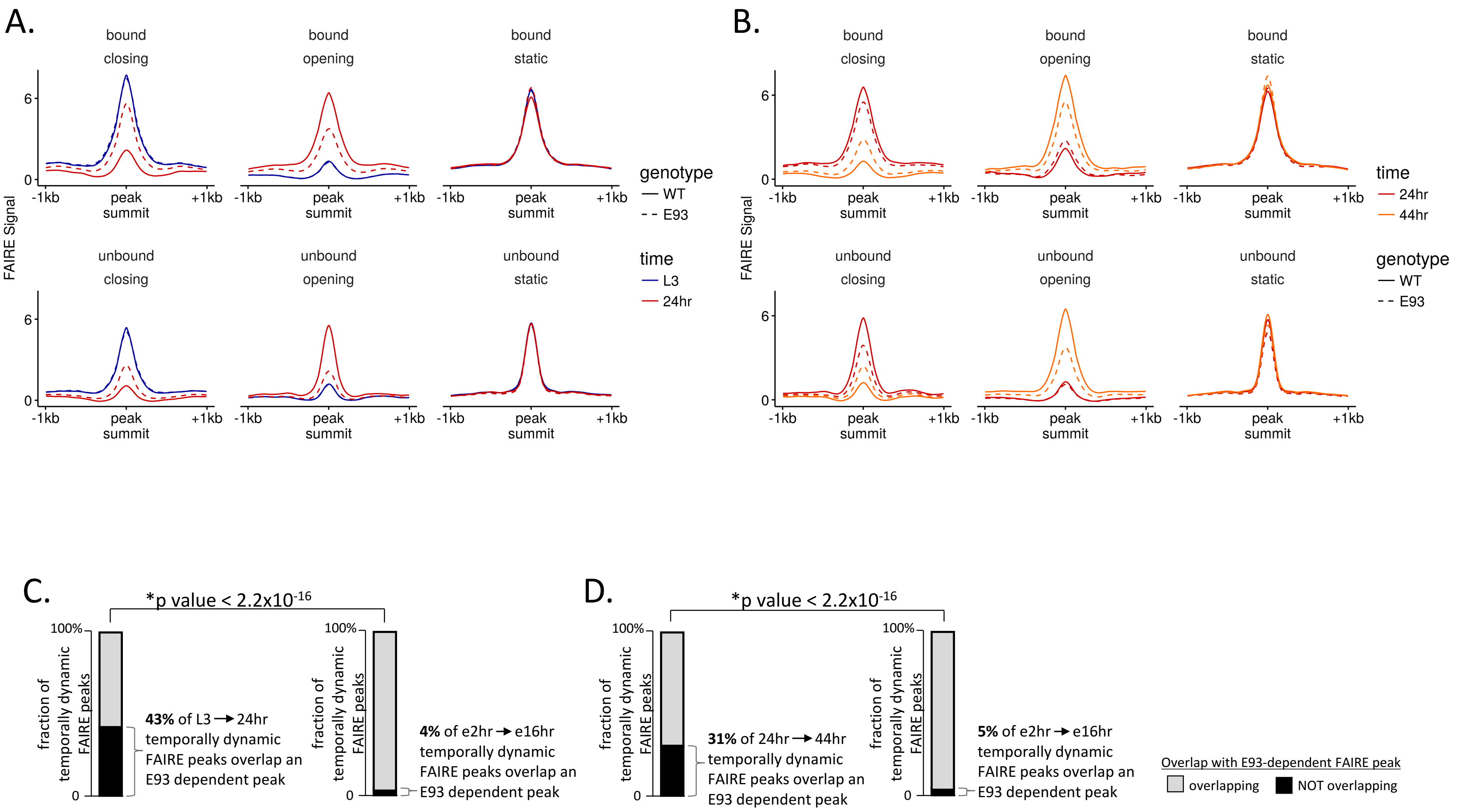

E93 mutant wings show heterochronic open chromatin defects

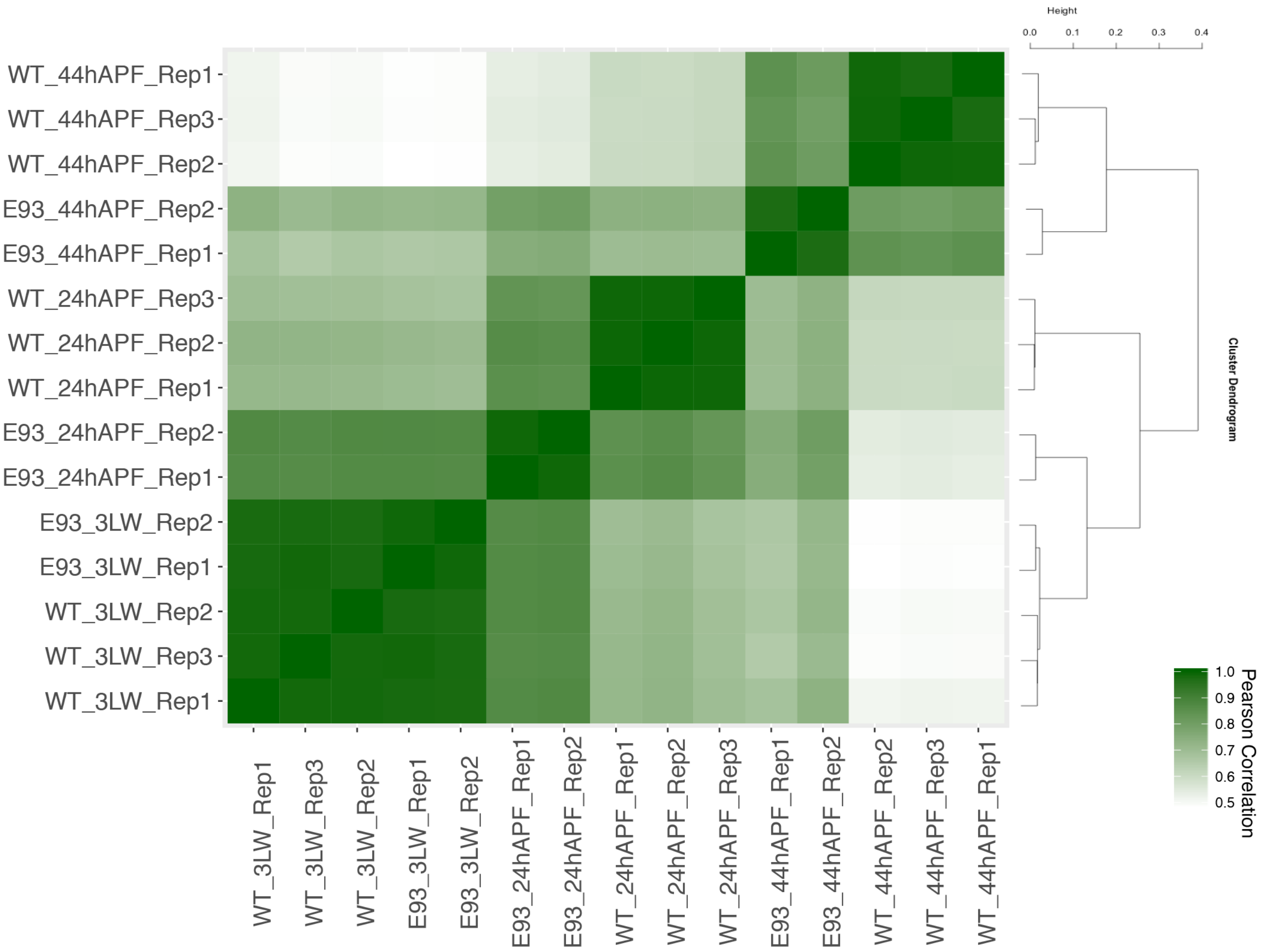

Supplement: Supplemental Material [file supp_gad.298182.117_Supplemental_Figures.pdf]
